# Food inflation, nutrition behavior, food insecurity, and anxiety: a Bayesian network analysis among Turkish adults

**DOI:** 10.3389/fnut.2026.1865375

**Published:** 2026-07-10

**Authors:** Hande Ongun Yilmaz, Sedat Arslan, Salim Yilmaz

**Affiliations:** 1 Department of Nutrition and Dietetics, Faculty of Health Sciences, Bandirma Onyedi Eylul University, Balikesir, Türkiye; 2 Department of Nutrition and Dietetics, Faculty of Health Sciences, Bursa Uludag University, Bursa, Türkiye; 3 Department of Health Management, Faculty of Health Sciences, Acibadem Mehmet Ali Aydinlar University, Istanbul, Türkiye; 4 Department of Health Management, Graduate School of Health Sciences, Acibadem Mehmet Ali Aydinlar University, Istanbul, Türkiye

**Keywords:** anxiety, Bayesian network, food inflation, food insecurity, nutrition behavior

## Abstract

**Background:**

Food inflation is a pervasive economic stressor that may disrupt nutritional behaviors and amplify food insecurity, yet the complex pathways linking these phenomena to psychological distress remain poorly understood. This study aimed to map the conditional dependency structure among food inflation-related behavioral changes, food insecurity, and anxiety in Turkish adults using Bayesian Network analysis.

**Methods:**

A cross-sectional online survey was conducted among 1,042 Turkish adults (72.9% female; 57.0% aged 18–24) between November and December 2025. The Effect of Food Inflation on Nutrition Behavior (EFINUB), The Impact of Food Inflation on Consumer Behavior (IFI-ConB), Food Insecurity Experience Scale, and Beck Anxiety Inventory were administered alongside sociodemographic measures. A Conditional Gaussian Bayesian Network with 23 nodes was learned using bootstrap-averaged structure learning (1,000 resamples, strength threshold ≥ 0.85), followed by Markov blanket analysis, conditional probability queries, and what-if policy simulations.

**Results:**

The network revealed 25 directed arcs organized into three isolated clusters: demographic, dietary habits, and scale-relationship. Food insecurity emerged as the sole member of the anxiety Markov blanket. Severe food insecurity rose from 1.2% to 48.2% (an absolute increase of 47.0 percentage points) across Low to High Food Consumption Impact groups. Simulating universal food security produced the largest anxiety reduction (−61.0%), whereas partial behavioral improvement reduced food insecurity but not anxiety. Sensitivity analyses confirmed structural robustness across threshold specifications (29 arcs at 0.70) and gender-balanced resampling (Jaccard = 0.808).

**Conclusion:**

Food insecurity is the primary conduit through which food inflation-related behavioral disruptions translate into anxiety. Policy responses may benefit from prioritizing direct food security support over partial behavioral assistance when the goal is to reduce anxiety burden.

## 1 Introduction

In recent years, rising inflation at the global level has led to substantial increases in the prices of essential goods, with food price inflation emerging as a critical economic issue directly affecting household living conditions. The rapid escalation in food prices constrains individuals' access to food, diminishes dietary quality, and exerts adverse effects on overall health. This process is particularly detrimental for vulnerable populations, increasing the risk of food insecurity and threatening sustainable access to adequate, safe, and nutritious food (1, 2).

Food insecurity extends beyond its impact on dietary quality and physical health, substantially affecting psychological wellbeing. A growing body of research has demonstrated strong and consistent associations between food insecurity and anxiety, depression, and psychological distress (3, 4). These associations are not solely driven by material deprivation but are also shaped by persistent uncertainty regarding food access, perceived loss of control, and concerns about the future (4, 5). Moreover, recent evidence suggests that inflation and rising living costs exacerbate psychological distress through increased food insecurity (6).

Within this context of economic stress, Türkiye represents a salient case, characterized in recent years by high inflation and pronounced increases in food prices. The pressure of rising food costs on household budgets elevates the risk of food insecurity and leads to notable changes in dietary behaviors. Evidence from Türkiye indicates an increasing prevalence of food insecurity among adults, which is negatively associated with psychological wellbeing (7). These patterns highlight that food inflation is not merely an economic phenomenon but a multidimensional process with behavioral and psychological consequences. According to the Turkish Statistical Institute (TurkStat), annual food inflation remained persistently elevated throughout the study period, reaching 28.31% for food and non-alcoholic beverages in December 2025, against an overall annual consumer price increase of 30.89%; food and non-alcoholic beverages account for roughly a quarter of the national consumer price basket (8). At the global level, the most recent estimates indicate that about 28% of the world's population experienced moderate or severe food insecurity in 2024 (2), and national evidence points to a rising burden of food insecurity among adults in Türkiye.

Existing literature has largely examined the relationships between inflation, food insecurity, and psychological health in a pairwise manner—for example, linking food insecurity to anxiety and depression (3, 4), or inflation to food insecurity (6)—while treating these associations in isolation. However, there is a notable lack of multivariate approaches that simultaneously capture their direct and indirect interrelationships within a single conditional dependency framework. This limitation hinders the identification of priority mechanisms for policy intervention. While many studies focus on the prevalence of food insecurity or its association with dietary behaviors, relatively few investigate these variables within a multivariate framework that accounts for conditional dependency structures alongside psychological outcomes (9). In reality, the effects of economic stress are not linear or unidirectional but instead reflect a complex interplay of behavioral, social, and psychological processes. Therefore, evaluating the simultaneous effects of inflation on dietary behavior, food insecurity, and psychological health within an integrated model represents a critical gap in the literature.

Conceptually, the constructs examined in this study can be organized along a stress-process sequence in which an economic stressor propagates through behavioral adaptation toward psychological strain. Food price inflation acts as an upstream macroeconomic stressor that first manifests as changes in nutritional and consumer behavior, captured here by the Effect of Food Inflation on Nutrition Behavior (EFINUB) and the Impact of Food Inflation on Consumer Behavior (IFI-ConB), as individuals adjust what they buy, how they shop, and what they eat in response to rising prices. These behavioral adaptations are theorized to translate into experienced food insecurity (FIES) when adjustments are insufficient to maintain adequate, reliable access to food. Food insecurity, in turn, is conceptualized as a proximal psychosocial stressor that elevates anxiety (BAI), consistent with stress-process and uncertainty-based models in which persistent threat to a basic need, rather than the distal economic shock itself, is the more immediate correlate of psychological distress (4, 5, 10). This framework motivates the expectation that the four instruments relate in an ordered, mediated fashion (behavioral impact to food insecurity to anxiety) rather than as independent or purely pairwise associations, and provides the conceptual rationale for examining their joint conditional dependency structure rather than isolated bivariate relationships.

The Bayesian Network approach has been increasingly employed in health research due to its capacity to uncover both direct and indirect relationships among variables in complex systems. By modeling conditional dependencies, this approach enables not only the identification of associations but also the assessment of their directionality and information flow (11). In this respect, Bayesian Networks provide a robust analytical framework for examining the multilayered relationships among inflation, food insecurity, and psychological health.

Given the limited number of studies simultaneously addressing the effects of food inflation on dietary behavior, food insecurity, and anxiety, the present research aims to elucidate the multivariate conditional dependency structure linking food inflation–related changes in dietary and consumer behaviors, food insecurity experiences, and anxiety among adults in Türkiye during a period of sustained food inflation, using Bayesian Network analysis. Unlike traditional regression models, the Bayesian Network approach simultaneously learns direct and indirect relationships among variables, characterizes the conditional independence structure of each variable, and allows probabilistic estimation of the potential outcomes of hypothetical intervention scenarios. Accordingly, the study addresses the following research questions: (i) What is the conditional dependency structure linking the impact of food inflation on dietary behavior with food insecurity and anxiety? (ii) Do demographic characteristics play a direct or indirect role within this structure? (iii) Which variable constitutes the most informative conditional dependency (i.e., the Markov blanket) of anxiety, and how strong is this association? (iv) How might hypothetical policy interventions (e.g., improving dietary behavior or directly ensuring food security) differentially affect food insecurity and anxiety?

## 2 Materials and methods

### 2.1 Study design and participants

This study employed a descriptive, cross-sectional, correlational design to examine the complex interrelationships among food inflation-related nutritional and consumer behavior changes, food insecurity, and anxiety in Turkish adults during a period of sustained food price inflation.

The target population comprised adults aged 18 years and older residing in Türkiye. A non-probability sampling strategy combining convenience and snowball techniques was employed due to the absence of a comprehensive sampling frame for the target population. Data were collected via an online self-administered questionnaire distributed through Google Forms between November and December 2025. The initial recruitment was conducted through social media platforms (WhatsApp, Facebook, Instagram, Twitter/X) and academic networks (convenience component), with respondents subsequently encouraged to share the survey link within their own networks (snowball component). To reduce duplicate submissions, the survey was configured to limit responses to one per Google account, and key items were set as required fields to minimize incomplete data. Data quality was further ensured through a structured post-collection screening protocol (Supplementary Table S2), in which responses with missing informed consent, missing or internally inconsistent entries (e.g., non-numeric or implausible age values), or out-of-scope residency were systematically excluded prior to analysis. No quota controls were applied during data collection; however, the resulting sample composition was compared against national population parameters to assess representativeness (see Supplementary Table S5). The initial dataset comprised 1,148 responses. After applying sequential exclusion criteria—non-consent (*n* = 30), missing or invalid age entries (*n* = 15), age below 18 years (*n* = 29), and non-residency in Türkiye (*n* = 23)—a total of 97 responses were excluded, yielding 1,051 cases. Subsequently, listwise deletion of 9 observations with missing BMI values produced a final analytic sample of 1,042 complete cases used for all analyses (see Supplementary Table S2 for the full exclusion flow diagram).

The sample size was determined by the survey's recruitment period and feasibility rather than by an a priori power analysis, which is not directly applicable to Bayesian Network structure learning; instead, the adequacy of the sample for recovering a stable dependency structure was established through bootstrap resampling (1,000 replicates), as described in Section 2.5.4.

### 2.2 Instruments

The survey instrument comprised sociodemographic questions and four validated psychometric scales.

**The Effect of Food Inflation on Nutrition Behavior (EFINUB):** The EFINUB was developed by Eşer Durmaz, Keser, and Ergenç (12) as a multidimensional instrument to assess the perceived impact of food inflation on nutritional behavior. The scale comprises 36 items across four subscales: Food Consumption (items 1–22), Food Insecurity (items 23–27), Panic Buying (items 28–33), and Budget (items 34–36). Each item is scored on a 5-point Likert scale (1 = Never to 5 = Always), yielding a total score range of 36–180. Higher scores indicate greater nutritional behavior disruption due to food inflation. The scale contains no reverse-scored items. The original development study reported a Cronbach's alpha of 0.974 (12). In the present sample, Cronbach's alpha was 0.984 for the total scale, with subscale alphas of 0.978 (Food Consumption), 0.924 (Food Insecurity), 0.941 (Panic Buying), and 0.880 (Budget), all exceeding both the original study values and the conventional 0.70 threshold.

**The Impact of Food Inflation on Consumer Behavior (IFI-ConB):** The IFI-ConB was developed by Haydaroglu and Bilgiç (13) to measure changes in consumer behavior attributable to food inflation. The scale comprises 17 items across three subscales: Food Consumption Pattern (items 1–6), Food Shopping Behaviors (items 7–12), and Food Purchasing Motives (items 13–17). Items are scored on a direction-independent format where “greatly increased” and “greatly decreased” both receive 3 points, “moderately increased/decreased” receive 2 points, “slightly increased/decreased” receive 1 point, and “no change” receives 0 points — reflecting that both directions of change represent inflation impact. The total score ranges from 0 to 51. The original development study reported a Cronbach's alpha of 0.92 (13). In the present sample, Cronbach's alpha was 0.962 for the total scale, with subscale alphas of 0.931 (Food Consumption Pattern), 0.940 (Food Shopping Behaviors), and 0.946 (Food Purchasing Motives), all exceeding the original study values.

**Food Insecurity Experience Scale (FIES):** The FIES was developed by the Food and Agriculture Organization (FAO) as part of the Voices of the Hungry project (14, 15) and has been validated in over 150 countries using the Gallup World Poll data. The Turkish adaptation, including validity and reliability assessment, was conducted by Kilinç et al. (7), who reported a Cronbach's alpha of 0.85. The scale comprises 8 items measuring the severity of food insecurity based on individuals' direct experiences during the past 12 months. Each item is scored dichotomously (Yes = 1, No = 0), yielding a total score range of 0–8. Established raw-score cutpoints classify respondents as food secure (0–3), moderately food insecure (4–6), or severely food insecure (7, 8); in the present analysis these raw-score categories were used as a simplified approximation rather than as prevalence estimates equated to the FAO global reference scale (see the Discussion). In the present sample, KR-20 reliability was 0.886, exceeding the Turkish adaptation value, and a Rasch measurement model confirmed adequate item fit and the expected item severity ordering (infit 0.83–1.16; Rasch reliability = 0.71; Supplementary Section S10, Supplementary Tables S11, S12 and Supplementary Figure S3).

**Beck Anxiety Inventory (BAI):** The BAI was developed by Beck, Epstein, Brown, and Steer (16) as a self-report measure of anxiety symptom severity, designed to discriminate anxiety from depression. The Turkish validity and reliability study was conducted by Ulusoy, Sahin, and Erkmen (17), who reported a Cronbach's alpha of 0.93 for the Turkish version. The scale comprises 21 items assessing somatic and cognitive anxiety symptoms, each scored on a 4-point scale (0 = Not at all to 3 = Severely), yielding a total score range of 0–63. Established clinical cutpoints classify anxiety severity as minimal (0–7), mild (8–15), moderate (16–25), or severe (26–63). In the present sample, Cronbach's alpha was 0.957, exceeding the original Turkish adaptation value.

### 2.3 Ethical approval and scale permissions

Ethical approval for this study was granted by the Bandirma Onyedi Eylül University Health Sciences Non-Interventional Research Ethics Committee (Decision Date: 16 October 2025). All procedures were conducted in accordance with the ethical standards of the institutional ethics committee and with the 1964 Helsinki Declaration and its later amendments. Informed consent was obtained from all participants prior to survey completion; participation was voluntary, and respondents could withdraw at any time without consequence.

Written permissions for use of the EFINUB were obtained from Eşer Durmaz, Keser, and Ergenç; permissions for the IFI-ConB were obtained from Haydaroglu and Bilgiç; the FIES is an open-access instrument developed by the FAO and were obtained from Kilinç; and the BAI Turkish version was used in accordance with published adaptation guidelines by Ulusoy, Sahin, and Erkmen (17). All scale permissions were documented and submitted to the ethics committee as part of the approval process.

### 2.4 Variable preparation

The Bayesian Network model incorporated 23 variables (nodes): 15 categorical and 8 continuous. Sociodemographic variables were categorized as follows: age was grouped into four categories (18–24, 25–34, 35–44, 45+); education was collapsed into three levels (below high school, high school, university and above); BMI was calculated from self-reported height and weight and classified as normal/underweight, overweight, or obese (underweight was merged with normal due to small cell size); income adequacy was assessed as a three-level perceived measure (income below expenses, equal to expenses, above expenses); breakfast habit was categorized as every morning, sometimes, or rarely/never (the latter combining “never” and “weekends only”); and daily meal count was categorized as 1, 2, 3, or 4+ meals. The complete variable list with definitions, types, and coding schemes is provided in Supplementary Table S1.

For the EFINUB Food Consumption subscale (items 1–22), a data-driven categorization was performed using Gaussian Mixture Models (GMM) to identify natural subgroups in the score distribution. A 3-component variable-variance model was selected based on BIC optimization, yielding three groups: Low impact (*n* = 131, 12.6%; score range 22–26, M = 23.4, SD = 1.5), Moderate impact (*n* = 660, 63.3%; score range 27–80, M = 55.9, SD = 14.9), and High impact (*n* = 251, 24.1%; score range 81–110, M = 94.0, SD = 9.0). The relative entropy of the classification was 0.7344, indicating acceptable classification certainty (18). GMM was evaluated for all seven continuous subscales, but only the EFINUB Food Consumption subscale yielded a tractable solution; the remaining six subscales produced 5–6 component solutions attributable to narrow score range artifacts and were therefore retained as continuous variables (see Supplementary Tables S3, S4).

The EFINUB Food Consumption subscale also served as the upstream behavioral-impact hub of the model, and its categorization into three levels (Low, Moderate, High) provided directly interpretable strata for the conditional probability queries and policy intervention scenarios, which require discrete intervention levels. The two outcome variables, FIES Food Insecurity (range 0–8) and BAI Anxiety (range 0–63), were retained as continuous nodes in the network; their established clinical cutpoints were not used to categorize them for structure learning or parameter estimation, but only to define outcome events at the conditional-probability query stage (Section 2.5.6).

### 2.5 Bayesian network analysis

#### 2.5.1 Theoretical framework

A Bayesian Network (BN) is a probabilistic graphical model that represents the joint probability distribution of a set of random variables *X* = {*X*_1_, *X*_2_, …, *X*_*n*_} as a directed acyclic graph (DAG) *G* = (*V, E*), where *V* denotes the set of nodes (variables) and *E* denotes the set of directed edges (arcs). The fundamental property of a BN is that the joint distribution factorizes according to the graph structure:


$$\begin{array}{l} P\left(X_{1},X_{2},\ldots ,X_{n}\right)=\overset{n}{\underset{i=1}{\prod }}P\left(X_{i}|Pa\left(X_{i}\right)\right) \end{array}$$

where *Pa*(*X*_*i*_) denotes the set of parent nodes of *X*_*i*_ in the DAG. Each variable is conditionally independent of its non-descendants given its parents, which is known as the local Markov property. This factorization enables efficient representation of high-dimensional joint distributions and, critically, allows identification of the directed conditional dependency structure among variables.

#### 2.5.2 Conditional gaussian parameterization

Because the present dataset contains both categorical (discrete) and continuous variables, a Conditional Gaussian (CG) Bayesian Network was employed. In a CG network, each continuous node *X*_*i*_ with discrete parents _*i*_ and continuous parents Γ_*i*_ follows a conditional Gaussian distribution:


$$\begin{array}{l} X_{i}|\Delta_{i}=\delta ,\Gamma_{i}=\gamma \mathrm{\sim N}\left(\mu_{i}\left(\delta \right)+\beta_{i}{\left(\delta \right)}^{⊤}\gamma ,\sigma_{i}^{2}\left(\delta \right)\right) \end{array}$$

where μ_*i*_(δ) is the conditional intercept for the discrete parent configuration δ, β_*i*_(δ) is the vector of regression coefficients on the continuous parents, and $\sigma_{i}^{2}\left(\delta \right)$ is the conditional variance. For discrete nodes, the conditional distributions are represented as conditional probability tables *P*(*X*_*i*_ = *x*_*i*_|*Pa*(*X*_*i*_) ).

The conditional Gaussian assumption was assessed by examining the distributional properties of the continuous nodes both marginally and conditionally on their parent configurations. Marginal skewness was modest for all continuous variables (|skewness| ≤ 0.77), and the residuals obtained after regressing each continuous node on its network parents showed skewness and excess kurtosis well within thresholds commonly regarded as acceptable for Gaussian-based models (|skewness| < 2, |excess kurtosis| < 7; observed maxima |skewness| ≤ 0.78 and |excess kurtosis| ≤ 2.28) (19, 20). Although Shapiro–Wilk tests were significant, an expected outcome at this sample size (*n* = 1,042), the magnitude of departure from normality was small, and conditional Gaussian networks are known to be robust to moderate non-normality of this kind (11) (see Supplementary Table S10).

#### 2.5.3 Structure learning

The BN structure was learned using score-based algorithms that search the space of possible DAGs to maximize a scoring function. The Bayesian Information Criterion adapted for Conditional Gaussian networks (BIC-CG) was used:


$$\begin{array}{l} BIC-CG\left(G,D\right)=\ln L\left(\hat{\theta }|G,D\right)-\frac{d}{2}\ln\left(n\right) \end{array}$$

where $L\left(\hat{\theta }|G,D\right)$ is the maximized likelihood of the data *D* given the graph *G* and maximum likelihood parameter estimates $\hat{\theta }$, *d* is the number of free parameters in the model, and *n* is the sample size. Two search algorithms were compared: Hill-Climbing (HC) and Tabu Search (tabu list size = 50). The Tabu Search algorithm was selected based on its superior BIC-CG score (−34,315.08 vs. −34,317.46), identifying 37 directed arcs.

#### 2.5.4 Bootstrap structural stability assessment

To evaluate the robustness of individual arcs, non-parametric bootstrap resampling was performed with *R* = 1, 000 iterations using the HC algorithm with BIC-CG scoring, parallelized across 14 CPU cores. Two stability metrics were computed for each candidate arc (*X*_*i*_→*X*_*j*_ ):

Bootstrap strength, defined as the proportion of bootstrap replicates in which an arc exists between *X*_*i*_ and *X*_*j*_ in either direction:


$$\begin{array}{l} S\left(X_{i},X_{j}\right)=\frac{1}{R}\overset{R}{\underset{r=1}{\sum }}𝟙\left[X_{i}X_{j}\in G^{\left(r\right)}\right] \end{array}$$

Direction probability, defined as the proportion of those replicates where the arc direction is from *X*_*i*_ to *X*_*j*_:


$$\begin{array}{l} D\left(X_{i}\to X_{j}\right)=\frac{\sum_{r=1}^{R}𝟙\left[X_{i}\to X_{j}\in G^{\left(r\right)}\right]}{\sum_{r=1}^{R}𝟙\left[X_{i}X_{j}\in G^{\left(r\right)}\right]} \end{array}$$

A model-averaged network was constructed by retaining arcs with *S* ≥ 0.85 and *D* ≥ 0.50, yielding a final structure of 25 directed arcs among 23 nodes. A supplementary model at threshold 0.70 (29 arcs) was also examined (see Supplementary Table S6; Supplementary Figure S1).

The threshold of 0.85 was chosen to be deliberately conservative relative to the data-driven optimum: the L1-based significance threshold computed automatically from the bootstrap strength distribution (21) was 0.503 (34 arcs), and adopting the higher value of 0.85 restricted the network to only the most reproducible arcs. This choice was further supported by the strongly bimodal distribution of bootstrap arc strengths, in which 354 candidate arcs fell below 0.50 and 50 fell at or above 0.85, with only 20 in the intermediate range, indicating a natural separation between stable and unstable arcs (Supplementary Figure S2).

#### 2.5.5 Markov blanket analysis

The Markov blanket of a target variable *X*_*i*_, denoted *MB*(*X*_*i*_), is defined as the minimal subset of variables that renders *X*_*i*_ conditionally independent of all remaining variables:


$$\begin{array}{l} X_{i}⊥⊥\left(V\backslash MB\left(X_{i}\right)\backslash \left\{X_{i}\right\}|MB\left(X_{i}\right)\right) \end{array}$$

The Markov blanket comprises the parents, children, and co-parents (other parents of the children) of *X*_*i*_:


$$\begin{array}{r} MB\left(X_{i}\right)=Pa\left(X_{i}\right)\cup Ch\left(X_{i}\right)\cup \{X_{j}:\exists X_{k}\in Ch\left(X_{i}\right),X_{j}\in Pa\left(X_{k}\right),\\ X_{j}\neq X_{i}\}. \end{array}$$

#### 2.5.6 Conditional probability queries

Probabilistic inference was performed to answer queries of the form *P*(*Event*∣*Evidence*). For categorical evidence variables, likelihood-weighted sampling (*N* = 50,000) was employed:


$$\begin{array}{l} P\left(Event|Evidence\right)\approx \frac{\sum_{i}^{N}w_{i}\cdot 𝟙\left[Event_{i}\right]}{\sum_{i}^{N}w_{i}} \end{array}$$

where $w_{i}=\prod_{k\in E}P\left(e_{k}|Pa{\left(X_{k}\right)}^{\left(i\right)}\right)$ is the importance weight for the *i*-th sample. *E* denotes the set of evidence nodes, and 𝟙[·] is the indicator function. For continuous variable conditioning (e.g., FIES score thresholds), empirical conditional probabilities were computed directly from the observed data, as likelihood weighting with continuous conditioning variables is computationally intractable in CG networks. All queries used a fixed random seed (2026) for reproducibility. Throughout, FIES and BAI remained continuous nodes in the fitted network; the cutpoints used in the queries (e.g., FIES > 6, BAI > 25) defined the target events for probabilistic inference rather than discretizing the variables themselves.

#### 2.5.7 What-if policy intervention analysis

The fitted BN was used to estimate the potential impact of hypothetical interventions on the outcome variables. Five counterfactual scenarios were constructed by manipulating the values of two key nodes—EFINUB Food Consumption Impact level and FIES Food Insecurity Experience—and estimating the resulting changes in the probabilities of severe food insecurity (FIES > 6) and severe anxiety (BAI > 25) relative to the observed sample baseline.

Two complementary estimation strategies were employed. For scenarios involving categorical intervention variables (Scenarios A and B), model-based likelihood-weighted sampling (*N* = 50,000) was used with the intervention variable clamped to the specified value, allowing the effects to propagate through the full conditional dependency structure of the learned DAG. For scenarios involving continuous variable interventions (Scenario C: FIES = 0) and within-group transitions (Scenarios D and E: High → Moderate, High → Low), empirical conditional probabilities were computed directly from the relevant subgroup data.

It should be noted that these scenario estimates represent model-implied probabilistic projections under hypothetical conditions rather than strict causal intervention effects. In the formal do-calculus framework (22), the identification of the interventional distribution requires satisfaction of the back-door criterion through a valid adjustment set *Z*:


$$\begin{array}{l} P\left(Y|do\left(X=x\right)\right)=\underset{z}{\sum }P\left(Y|X=x,\;Z=z\right)\;P\left(Z=z\right). \end{array}$$

In observational cross-sectional data, these graphical identification conditions cannot be formally verified in the absence of experimental manipulation, and unmeasured confounding cannot be ruled out. Accordingly, the scenario estimates should be interpreted as indicative probabilistic contrasts— *P*(*Y*|*X* = *x*) rather than *P*(*Y*|*do*(*X* = *x*))–that illustrate the magnitude and direction of potential changes implied by the learned network structure, rather than as unbiased estimates of causal intervention effects.

#### 2.5.8 Sensitivity analysis

Three sensitivity analyses were conducted to evaluate the robustness of the primary findings.

- Bootstrap threshold sensitivity was examined by constructing a supplementary network at a relaxed strength threshold of S ≥ 0.70 (in addition to the primary S ≥ 0.85), yielding 29 directed arcs compared to 25 in the primary model. The distribution of bootstrap arc strength values across all candidate arcs was inspected, and the number of retained arcs was computed at seven candidate thresholds (0.50, 0.60, 0.70, 0.80, 0.85, 0.90, 0.95) to evaluate the sensitivity of the network structure to threshold selection (Supplementary Figures S1, S2; Supplementary Table S6).
- The stability of the GMM-based categorization of the EFINUB Food Consumption subscale was assessed by comparing model fit indices across 1- to 6-component solutions under varying variance structures. The optimal 3-component variable-variance model was selected based on BIC and ICL criteria, and classification certainty was evaluated using relative entropy (see Supplementary Tables S3, S4).
- A gender-balanced subsample (*n* = 564; 282 males, 282 females) was created by random undersampling to evaluate whether the network structure was driven by the gender composition of the original sample. The full BN learning and bootstrap stability procedure was repeated on this subsample, and structural agreement between the full-sample and balanced-sample networks was quantified using the Jaccard similarity index:

$$\begin{array}{l} J\left(A,B\right)=\frac{\left|A\cap B\right|}{\left|A\cup B\right|} \end{array}$$

where *A* and *B* are the sets of directed arcs in the two networks.

### 2.6 Data analysis

Data management, cleaning, and all statistical analyses were performed using R version 4.5.2 [(23); complete package versions are provided in Supplementary Table S8]. Sociodemographic characteristics were compared across EFINUB food consumption impact groups (Low, Moderate, High) using Pearson's chi-square test for categorical variables (after verifying that fewer than 20% of expected cell frequencies fell below 5) and Kruskal-Wallis H test for continuous variables (as Shapiro-Wilk tests indicated non-normal distributions in all groups, all *p* < 0.001). When significant omnibus differences were detected, *post-hoc* pairwise comparisons were conducted using Bonferroni-corrected Wilcoxon rank-sum tests. Effect sizes were estimated using epsilon-squared (ε^2^) for Kruskal-Wallis tests. Internal consistency reliability was assessed using Cronbach's alpha for Likert-scaled instruments and Kuder-Richardson Formula 20 (KR-20) for the dichotomously scored FIES, computed via the psych package (24). For the FIES specifically, a Rasch (one-parameter logistic) measurement model was additionally fitted to the eight dichotomous items by conditional maximum likelihood using the RM weights package (25), following FAO guidance for FIES data analysis and established item response theory principles for food security measurement (26, 55); item severity parameters, infit and outfit fit statistics, Rasch reliability, and respondent severity by raw score are reported in Supplementary Section S10. The sample-specific Rasch metric was not equated to the FAO global reference scale; accordingly, the FIES raw-score categories are reported as a simplified approximation and not as internationally calibrated prevalence estimates. Gaussian Mixture Model analysis for data-driven categorization was performed using the mclust package (27), evaluating models with 1 to 6 components and selecting the optimal solution based on BIC and ICL criteria.

Bayesian Network structure learning, bootstrap stability analysis, parameter estimation, Markov blanket identification, and conditional probability queries were performed using the bnlearn package (11, 28). Bootstrap resampling (1,000 iterations) was parallelized across 14 CPU cores using the parallel package. Network visualization was produced using the igraph (29, 30), ggraph (31), and ggplot2 (32) packages, with multi-panel figure composition via patchwork. Statistical significance was evaluated at α = 0.05 for all inferential tests. All reported *p*-values are two-sided unless otherwise noted.

## 3 Results

### 3.1 Sample characteristics

The final analytic sample comprised 1,042 adults (72.9% female; mean age concentrated in the 18–24 group, 57.0%). The majority were university-educated (80.6%), single (73.6%), and unemployed (57.8%). The perceived income distribution showed that 24.4% reported income below expenses, 56.7% equal to expenses, and 18.9% above expenses. Regarding health characteristics, 11.4% reported a diagnosed chronic disease, 61.7% had normal BMI, 27.2% were overweight, and 11.1% were obese. Lifestyle variables indicated that 54.8% engaged in regular physical activity, 35.9% were current smokers, and 10.1% consumed alcohol. Dietary habits revealed that 51.9% ate breakfast every morning, while 45.2% reported regularly skipping meals. Sample representativeness relative to the national population is discussed in Supplementary Table S5.

Table 1 presents the sociodemographic and health characteristics of the study sample stratified by GMM-derived Food Consumption Impact level. Statistically significant differences across the three EFINUB groups were observed for gender (χ^2^ = 12.98, df = 2, *p* = 0.002), income adequacy (χ^2^ = 41.83, df = 4, *p* < 0.001), education (χ^2^ = 15.54, df = 4, *p* = 0.004), employment status (χ^2^ = 11.63, df = 2, *p* = 0.003), BMI category (χ^2^ = 10.02, df = 4, *p* = 0.040), smoking (χ^2^ = 8.83, df = 2, *p* = 0.012), alcohol use (χ^2^ = 8.10, df = 2, *p* = 0.017), and meals per day (χ^2^ = 14.49, df = 6, *p* = 0.025). No significant differences were found for age group (*p* = 0.288), chronic disease (*p* = 0.906), marital status (*p* = 0.609), physical activity (*p* = 0.619), breakfast habit (*p* = 0.173), or meal skipping (*p* = 0.064).

**Table 1:** Sociodemographic and health characteristics of the study sample by food consumption impact level (*n* = 1,042).

Variable	Total (*n* = 1,042)	Low (*n* = 131)	Moderate (*n* = 660)	High (*n* = 251)	Test statistic	*p*
**Gender**					χ^2^ = 12.98	**0.002**
Male	282 (27.1%)	37 (28.2%)	156 (23.6%)	89 (35.5%)		
Female	760 (72.9%)	94 (71.8%)	504 (76.4%)	162 (64.5%)		
**Age group**					χ^2^ = 7.37	0.288
18–24	594 (57.0%)	65 (49.6%)	388 (58.8%)	141 (56.2%)		
25–34	234 (22.5%)	39 (29.8%)	141 (21.4%)	54 (21.5%)		
35–44	108 (10.4%)	12 (9.2%)	64 (9.7%)	32 (12.7%)		
45+	106 (10.2%)	15 (11.5%)	67 (10.2%)	24 (9.6%)		
**Income adequacy**					χ^2^ = 41.83	**< 0.001**
Below expenses	254 (24.4%)	20 (15.3%)	141 (21.4%)	93 (37.1%)		
Equal to expenses	591 (56.7%)	70 (53.4%)	399 (60.5%)	122 (48.6%)		
Above expenses	197 (18.9%)	41 (31.3%)	120 (18.2%)	36 (14.3%)		
**Education**					χ^2^ = 15.54	**0.004**
Below high school	55 (5.3%)	6 (4.6%)	27 (4.1%)	22 (8.8%)		
High school	147 (14.1%)	18 (13.7%)	82 (12.4%)	47 (18.7%)		
University+	840 (80.6%)	107 (81.7%)	551 (83.5%)	182 (72.5%)		
**Chronic disease**	119 (11.4%)	16 (12.2%)	76 (11.5%)	27 (10.8%)	χ^2^ = 0.20	0.906
**Marital status**					χ^2^ = 0.99	0.609
Single	767 (73.6%)	92 (70.2%)	491 (74.4%)	184 (73.3%)		
Married	275 (26.4%)	39 (29.8%)	169 (25.6%)	67 (26.7%)		
**Employment**					χ^2^ = 11.63	**0.003**
Employed	440 (42.2%)	72 (55.0%)	258 (39.1%)	110 (43.8%)		
Unemployed	602 (57.8%)	59 (45.0%)	402 (60.9%)	141 (56.2%)		
**BMI category**					χ^2^ = 10.02	**0.040**
Normal/Underweight	643 (61.7%)	87 (66.4%)	419 (63.5%)	137 (54.6%)		
Overweight	283 (27.2%)	30 (22.9%)	166 (25.2%)	87 (34.7%)		
Obese	116 (11.1%)	14 (10.7%)	75 (11.4%)	27 (10.8%)		
**Physical activity**	571 (54.8%)	77 (58.8%)	358 (54.2%)	136 (54.2%)	χ^2^ = 0.96	0.619
**Smoking**	374 (35.9%)	48 (36.6%)	217 (32.9%)	109 (43.4%)	χ^2^ = 8.83	**0.012**
**Alcohol use**	105 (10.1%)	10 (7.6%)	58 (8.8%)	37 (14.7%)	χ^2^ = 8.10	**0.017**
**Breakfast habit**					χ^2^ = 6.37	0.173
Every morning	541 (51.9%)	80 (61.1%)	331 (50.2%)	130 (51.8%)		
Sometimes	363 (34.8%)	34 (26.0%)	243 (36.8%)	86 (34.3%)		
Rarely/Never	138 (13.2%)	17 (13.0%)	86 (13.0%)	35 (13.9%)		
**Meals per day**					χ^2^ = 14.49	**0.025**
1	26 (2.5%)	3 (2.3%)	11 (1.7%)	12 (4.8%)		
2	552 (53.0%)	70 (53.4%)	363 (55.0%)	119 (47.4%)		
3	370 (35.5%)	49 (37.4%)	233 (35.3%)	88 (35.1%)		
4+	94 (9.0%)	9 (6.9%)	53 (8.0%)	32 (12.7%)		
**Meal skipping**					χ^2^ = 8.90	0.064
No	197 (18.9%)	34 (26.0%)	117 (17.7%)	46 (18.3%)		
Sometimes	374 (35.9%)	51 (38.9%)	241 (36.5%)	82 (32.7%)		
Yes	471 (45.2%)	46 (35.1%)	302 (45.8%)	123 (49.0%)		
**FIES**					H = 293.62	**< 0.001**
(M ± SD)	3.54 ± 2.91	0.78 ± 1.62	3.20 ± 2.65	5.89 ± 2.27		
Q_2_ (Q_1_-Q_3_)	4.0 (0.0–6.0)	0.0 (0.0–1.0)	3.0 (1.0–5.0)	6.0 (4.0–8.0)		
**BAI**					H = 68.53	**< 0.001**
(M ± SD)	16.21 ± 14.22	8.40 ± 11.95	16.52 ± 14.24	19.47 ± 13.86		
Q_2_ (Q_1_-Q_3_)	14.0 (3.0–25.0)	4.0 (0.0–11.5)	14.0 (3.8–26.0)	19.0 (8.0–28.0)		
**EFINUB food insec**.					H = 611.34	**< 0.001**
(M ± SD)	14.78 ± 5.90	6.11 ± 1.44	13.93 ± 4.29	21.55 ± 2.94		
Q_2_ (Q_1_-Q_3_)	15.0 (10.0–20.0)	6.0 (5.0–7.0)	15.0 (10.8–16.0)	21.0 (20.0–25.0)		
**EFINUB panic buy**.					H = 490.15	**< 0.001**
(M ± SD)	18.61 ± 6.95	9.34 ± 4.26	17.77 ± 5.56	25.65 ± 3.83		
Q_2_ (Q_1_-Q_3_)	18.0 (13.0–24.0)	8.0 (6.0–11.5)	18.0 (14.0–21.0)	26.0 (24.0–30.0)		
**EFINUB budget**					H = 441.60	**< 0.001**
(M ± SD)	7.86 ± 3.71	3.77 ± 1.71	7.22 ± 2.95	11.69 ± 2.90		
Q_2_ (Q_1_-Q_3_)	8.0 (4.2–11.0)	3.0 (3.0–3.0)	7.0 (5.0–9.0)	12.0 (10.0–14.0)		
**IFI-ConB food cons. pattern**					H = 396.86	**< 0.001**
(M ± SD)	6.18 ± 5.26	1.16 ± 2.98	5.13 ± 4.08	11.54 ± 4.72		
Q_2_ (Q_1_-Q_3_)	6.0 (1.0–10.0)	0.0 (0.0–1.0)	5.0 (2.0–7.0)	12.0 (8.0–15.0)		
**IFI-ConB food shop. behav**.					H = 297.51	**< 0.001**
(M ± SD)	8.22 ± 5.68	2.53 ± 3.96	7.63 ± 5.06	12.76 ± 4.53		
Q_2_ (Q_1_-Q_3_)	7.5 (4.0–12.0)	0.0 (0.0–4.0)	6.0 (4.0–12.0)	13.0 (10.0–17.0)		
**IFI-ConB food purch. motives**					H = 256.88	**< 0.001**
(M ± SD)	6.13 ± 4.84	2.50 ± 4.22	5.37 ± 4.31	10.03 ± 4.00		
Q_2_ (Q_1_-Q_3_)	5.0 (1.0–10.0)	0.0 (0.0–3.5)	5.0 (1.0–9.0)	10.0 (8.0–13.0)		

Values are *n* (%) for categorical variables and both M ± SD and median with interquartile range [Q_2_ (Q1–Q3)] for continuous variables. Low, Moderate, and High groups were derived from Gaussian Mixture Model classification of the EFINUB Food Consumption subscale (items 1–22). χ^2^ = Pearson's chi-square test (all expected cell frequencies ≥ 5 verified); H = Kruskal-Wallis H test (Shapiro-Wilk tests indicated non-normality in all groups, *p* < 0.001). All pairwise *post-hoc* comparisons for continuous variables were significant (Bonferroni-corrected Wilcoxon, *p* < 0.005). Bold *p*-values indicate statistical significance at α = 0.05. FIES, Food Insecurity Experience Scale; BAI, Beck Anxiety Inventory; EFINUB, The Effect of Food Inflation on Nutrition Behavior; IFI-ConB, The Impact of Food Inflation on Consumer Behavior.

All continuous outcome variables differed significantly across the three EFINUB groups (Kruskal-Wallis, all *p* < 0.001). FIES scores showed the strongest differentiation (H = 293.62, df = 2, ε^2^ = 0.281), increasing from M = 0.78 (SD = 1.62) in the Low group to M = 5.89 (SD = 2.27) in the High group. BAI scores also increased monotonically (H = 68.53, df = 2, ε^2^ = 0.064), from M = 8.40 (SD = 11.95) to M = 19.47 (SD = 13.86). All EFINUB and IFIConB subscale scores showed large effect sizes (ε^2^ ranging from 0.245 to 0.587) with significant *post-hoc* pairwise comparisons between all three groups (Bonferroni-corrected Wilcoxon, all *p* < 0.005) (Table 1).

**Figure 1:**
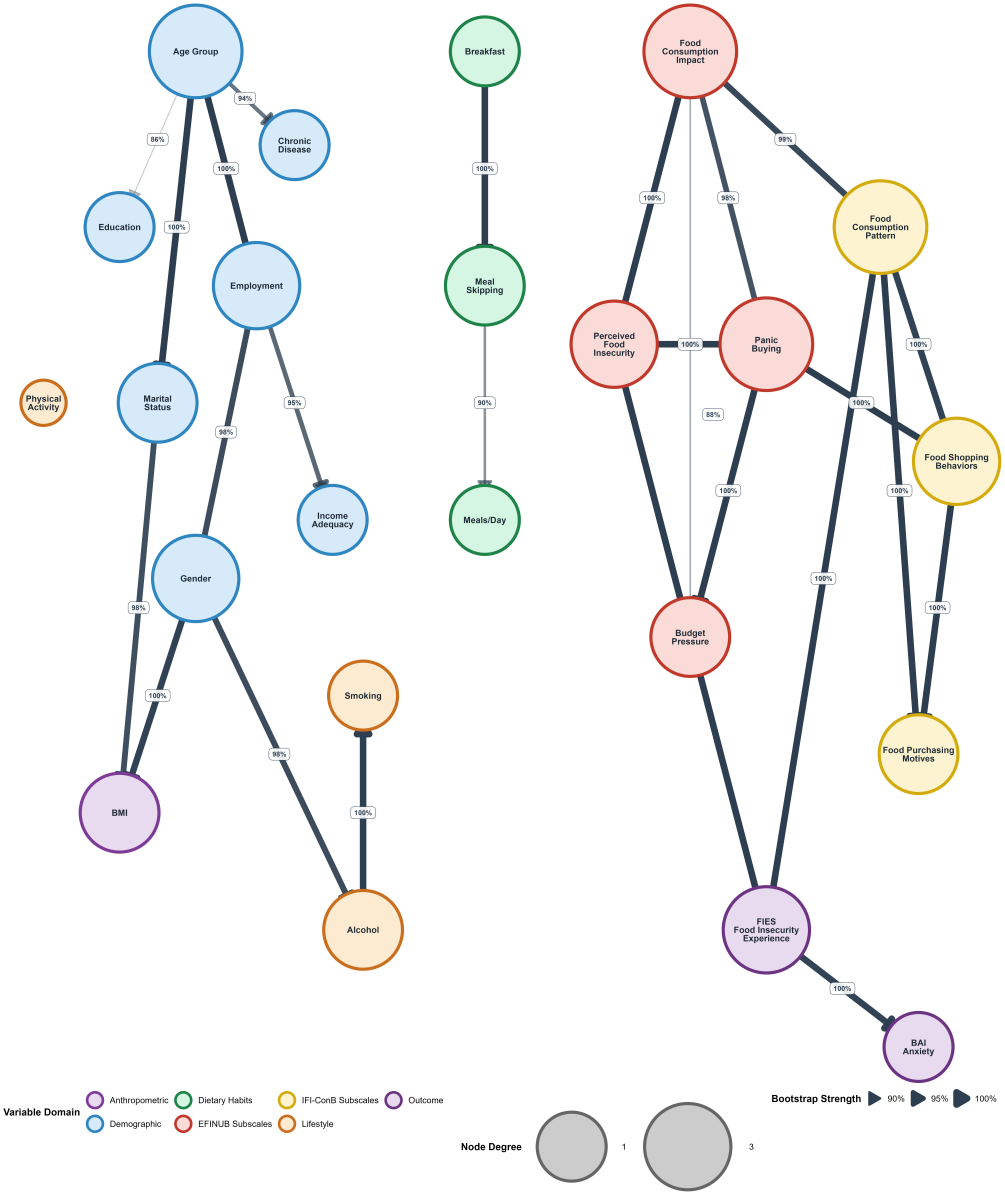
Bootstrap-averaged Bayesian Network structure learned from 1,042 Turkish adults (strength threshold S ≥ 0.85, direction threshold D ≥ 0.50). Nodes (*n* = 23) are colored by domain: demographic (blue), anthropometric (purple), lifestyle (orange), dietary habits (green), EFINUB subscales (red), IFI-ConB subscales (yellow), and outcome (violet). Node size is proportional to total degree; edge width and opacity reflect bootstrap arc strength (1,000 resamples), with labels showing the proportion of replicates in which each arc appeared, and arrows the predominant direction. The network organized into three clusters—demographic, dietary habits, and scale-relationship—the last converging on FIES Food Insecurity and BAI Anxiety as terminal nodes.

The bootstrap-averaged BN (threshold = 0.85) revealed 25 directed arcs among 23 nodes, organizing into three distinct clusters with limited inter-cluster connectivity (Figure 1).

**Demographic cluster:** Age group served as the central hub, with directed arcs to education (S = 0.86, D = 0.55), chronic disease (S = 0.94, D = 0.62), marital status (S = 1.00, D = 0.59), and employment (S = 1.00, D = 0.55). Employment subsequently influenced gender (S = 0.98, D = 0.66) and income adequacy (S = 0.95, D = 0.68). Gender directed arcs to BMI (S = 1.00, D = 0.85) and alcohol use (S = 0.98, D = 0.61), while marital status directed to BMI (S = 0.98, D = 0.89). Alcohol directed to smoking (S = 1.00, D = 0.68). This cluster showed no direct arcs to the scale-relationship cluster at the 0.85 threshold — the food inflation impact variables are conditionally independent of demographic variables given the absence of connecting arcs.

**Dietary habits cluster:** Breakfast habit directed to meal skipping (S = 1.00, D = 0.57), which directed to meals per day (S = 0.92, D = 0.64), forming a simple Markov chain. This cluster was isolated from both other clusters.

**Scale-relationship cluster:** The EFINUB Food Consumption Impact node emerged as the central hub with four outgoing arcs, all achieving D = 1.00: to Perceived Food Insecurity (S = 1.00), Panic Buying (S = 0.98), Budget Pressure (S = 0.88), and Food Consumption Pattern (S = 0.99). Beyond these four direct outgoing arcs, the learned DAG also included downstream dependencies among the remaining scale and outcome variables: Perceived Food Insecurity directed to both Panic Buying (S = 1.00, D = 0.63) and FIES Food Insecurity (S = 1.00, D = 0.92); Panic Buying directed to Budget Pressure (S = 1.00, D = 0.86) and Food Shopping Behaviors (S = 1.00, D = 0.97); Food Consumption Pattern directed to Food Shopping Behaviors (S = 1.00, D = 0.75), Food Purchasing Motives (S = 1.00, D = 0.79), and FIES Food Insecurity (S = 1.00, D = 0.70); Food Shopping Behaviors directed to Food Purchasing Motives (S = 1.00, D = 0.67); and FIES Food Insecurity directed to BAI Anxiety (S = 1.00, D = 0.90).

Following the learned structure, the joint distribution of the scale-relationship cluster factorizes as:


$$\begin{array}{l} P(EFINUB_{BT},EFINUB_{FI},EFINUB_{PB},EFINUB_{BU},IFIConB_{FC},\\ IFIConB_{GS},IFIConB_{PM},FIES,BAI)=P\left(EFINUB_{BT}\right)\\ P\left(EFINUB_{FI}|EFINUB_{BT}\right)\times P(EFINUB_{PB}|EFINUB_{BT},\\ EFINUB_{FI})\times P\left(EFINUB_{BU}|EFINUB_{BT},EFINUB_{PB}\right)\\ P\left(IFIConB_{FC}|EFINUB_{BT}\right)\;\times P(IFIConB_{GS}|EFINUB_{PB},\\ IFIConB_{FC})P\left(IFIConB_{PM}|IFIConB_{FC},IFIConB_{GS}\right)\\ \times P\left(FIES|EFINUB_{FI},IFIConB_{FC}\right)P\left(BAI|FIES\right). \end{array}$$

This factorization reveals the primary pathway: EFINUB_BT_ → EFINUB_FI_ → FIES → BAI. Critically, BAI appears only as a child of FIES with no other parents or children in the DAG, rendering it conditionally independent of all other 21 variables given FIES alone. The complete arc strength table is presented in Table 2. A supplementary network at threshold 0.70 is discussed in Section 5 of the Supplementary Material.

**Table 2:** Bootstrap arc strength and direction probability for the averaged Bayesian network (threshold = 0.85).

From	To	Strength	Direction
EFINUB food consumption impact	Perceived food insecurity	1.000	1.000
EFINUB food consumption impact	Panic buying	0.976	1.000
EFINUB food consumption impact	Budget pressure	0.878	1.000
EFINUB food consumption impact	Food consumption pattern	0.988	1.000
Perceived food insecurity	Panic buying	1.000	0.628
Perceived food insecurity	FIES food insecurity	1.000	0.921
Panic buying	Budget pressure	1.000	0.863
Panic buying	Food shopping behaviors	1.000	0.967
Food consumption pattern	Food shopping behaviors	0.996	0.747
Food consumption pattern	Food purchasing motives	1.000	0.789
Food consumption pattern	FIES food insecurity	1.000	0.698
Food shopping behaviors	Food purchasing motives	1.000	0.669
FIES food insecurity	BAI Anxiety	0.995	0.904
Age group	Education	0.860	0.554
Age group	Chronic disease	0.938	0.620
Age group	Marital status	1.000	0.590
Age group	Employment	1.000	0.545
Employment	Gender	0.984	0.657
Employment	Income adequacy	0.953	0.680
Gender	BMI	0.996	0.850
Gender	Alcohol	0.984	0.612
Marital status	BMI	0.981	0.885
Alcohol	Smoking	0.999	0.676
Breakfast	Meal skipping	1.000	0.559
Meal skipping	Meals per day	0.904	0.623

Strength = proportion of 1,000 bootstrap replicates in which the arc appeared; Direction = proportion of those replicates in which the arc direction was as shown. Arcs are ordered by domain: EFINUB/IFI-ConB scale relationships first, then demographic, then dietary habits.

The bootstrap arc strength and direction values presented in Table 2 revealed that the strongest and most directionally consistent connections in the network were concentrated between the EFINUB subscales and the outcome variables.

All four arcs emanating from EFINUB Food Consumption Impact toward Perceived Food Insecurity, Panic Buying, Food Consumption Pattern, and Budget Pressure exhibited perfect directional certainty (direction = 1.000), which suggested that the level of Food Consumption Impact served as a central hub within the network. Similarly, the Panic Buying → Food Shopping Behaviors (strength = 1.000, direction = 0.967) and FIES Food Insecurity → BAI Anxiety (strength = 0.995, direction = 0.904) connections demonstrated high bootstrap consistency, indicating that, within the network, higher food inflation impact was associated with changes in shopping patterns through panic buying behavior and with elevated anxiety through the food insecurity experience pathway. The connections among demographic variables, despite their high strength values, displayed relatively low direction probabilities (e.g., Age Group → Education: 0.554; Age Group → Employment: 0.545), which suggested that the causal direction remained ambiguous in these relationships and that bidirectional dependencies may have been present. In contrast, the Gender → BMI (direction = 0.850) and Marital Status → BMI (direction = 0.885) connections pointed to a clearer directional structure in the conditional dependencies between these demographic variables and the anthropometric variable (Table 2).

**Figure 2:**
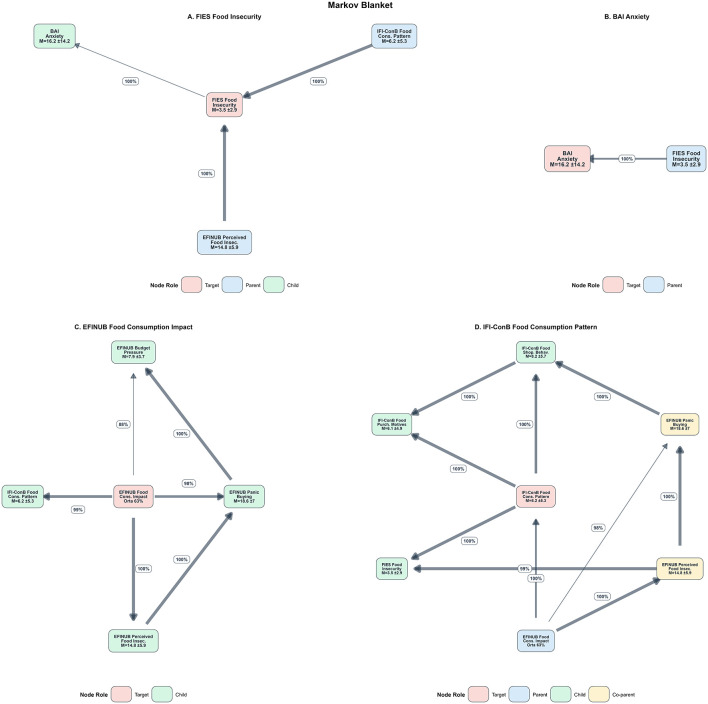
Markov blanket subgraphs for four key variables in the bootstrap-averaged Bayesian Network. **(A)** FIES Food Insecurity Experience: parents = Perceived Food Insecurity, Food Consumption Pattern; child = BAI Anxiety. **(B)** BAI Anxiety: parent = FIES Food Insecurity (single-node Markov blanket). **(C)** EFINUB Food Consumption Impact: children = Perceived Food Insecurity, Panic Buying, Budget Pressure, Food Consumption Pattern (root node with no parents). **(D)** IFI-ConB Food Consumption Pattern: parent = EFINUB Food Consumption Impact; children = Food Shopping Behaviors, Food Purchasing Motives, FIES Food Insecurity; co-parents = Panic Buying, Food Shopping Behaviors. Node colors indicate structural role: target (red), parent (blue), child (green), and co-parent (yellow). Edge labels show bootstrap arc strength. Descriptive statistics (mode for categorical, M ± SD for continuous variables) are displayed within each node.

Markov blanket analysis identified the minimal sufficient predictor sets for the key outcome variables (Figure 2).

The Markov blanket of FIES comprised three nodes: *MB*(*FIES*) = {*EFINUB*_*FI*_, *IFIConB*_*FC*_, *BAI*}, where EFINUB_FI_ and IFI-ConB_FC_ are parents and BAI is the sole child. The Markov blanket of BAI Anxiety contained a single node: *MB*(*BAI*) = {*FIES*}. Within the learned network and at the selected bootstrap threshold, this implies that—conditional on food insecurity—none of the remaining 21 nodes provided additional information about anxiety severity. This is a property of the estimated model rather than a definitive feature of the real-world system; it should be interpreted as a conditional-independence relationship within the present network rather than as evidence that no other factors influence anxiety. Notably, this single-node Markov blanket was stable across bootstrap thresholds (0.70–0.90) and was reproduced when food insecurity was modeled as a continuous variable (Supplementary Table S9), indicating that the result is robust to key analytical choices even though it remains model-dependent. The Markov blanket of EFINUB Food Consumption Impact comprised four child nodes: *MB*(*EFINUB*_*BT*_) = {*EFINUB*_*FI*_, *EFINUB*_*PB*_, *EFINUB*_*BU*_, *IFIConB*_*FC*_}, confirming its role as the upstream hub with no parents (a root node in the DAG) (Figure 2).

**Figure 3:**
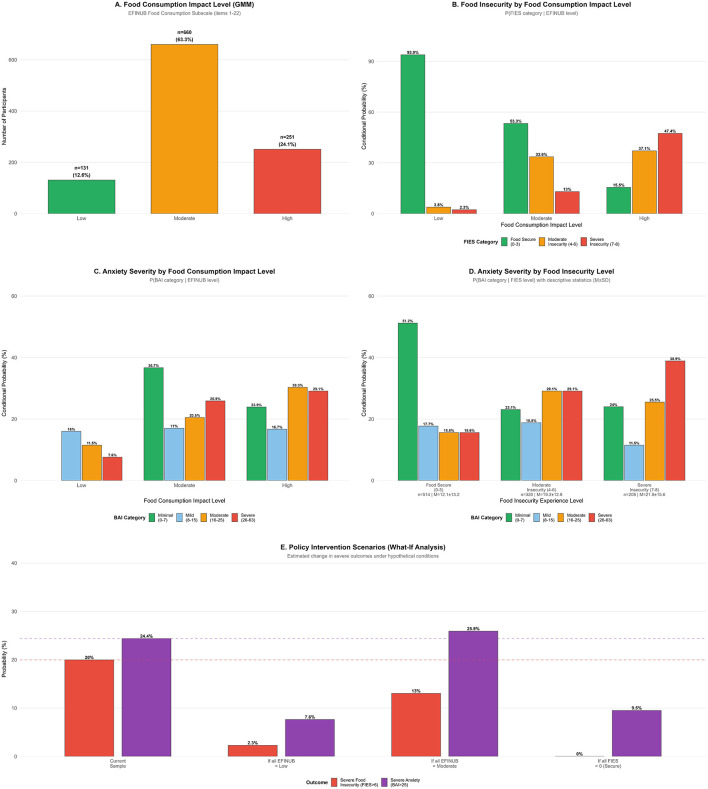
Conditional probability distributions and policy intervention scenarios derived from the fitted Bayesian Network. **(A)** Distribution across GMM-identified Food Consumption Impact levels (Low, Moderate, High). **(B)** Probability of food insecurity severity given Food Consumption Impact level (severe food insecurity rising from 1.2% to 48.2%, Low to High). **(C)** Probability of anxiety severity given Food Consumption Impact level. **(D)** Probability of anxiety severity given FIES level, with n and M ± SD per category. **(E)** What-if scenarios comparing severe food insecurity (FIES > 6) and severe anxiety (BAI > 25) against the sample baseline (dashed lines). Probabilities were estimated by likelihood-weighted sampling (N = 50,000) for categorical conditioning and empirical proportions for continuous thresholds.

Conditional probability queries derived from the fitted Bayesian Network revealed strong dose–response relationships along the primary network pathway (Figure 3).

The pathway from Food Consumption Impact to food insecurity demonstrated a pronounced gradient (Figure 3, Panel B). As EFINUB impact level increased from Low to High, the conditional probability of food security (FIES ≤ 3) declined from 83.0% to 11.6%, whereas severe food insecurity (FIES > 6) rose from 1.2% to 48.2%—an absolute increase of 47.0 percentage points. Although this corresponds to an approximately 40-fold relative increase, we emphasize the absolute difference because the low baseline prevalence in the Low group (1.2%) makes the fold-change ratio sensitive to small fluctuations and prone to overstating precision. The absolute risk difference indicates that individuals in the High impact group were substantially more likely to experience severe food insecurity than their Low impact counterparts.

A similar, albeit less pronounced, gradient was observed for the Food Consumption Impact to anxiety pathway (Figure 3, Panel C). The conditional probability of severe anxiety (BAI > 25) increased from 17.6% in the Low group to 36.0% in the High group, an absolute increase of 18.4 percentage points (an approximately 2-fold relative increase). When moderate and severe categories were combined (BAI > 15), the cumulative probability rose from 41.6% to 63.6%, suggesting that the psychological burden of food inflation-related behavioral changes extended beyond the most extreme cases.

The direct relationship between food insecurity and anxiety severity was further examined through empirical conditional probability analysis across FIES severity levels (Figure 3, Panel D). The descriptive statistics accompanying each FIES category confirmed a monotonic increase in mean BAI scores as food insecurity deepened, with detailed conditional distributions presented in Table 3.

**Table 3:** Conditional probability of anxiety severity by food insecurity level (empirical estimates).

FIES level	*n*	BAI M ±SD	Median	*P* (BAI ≤ 7)	*P* (BAI > 15)	*P* (BAI > 25)
0 (full security)	263	9.63 ± 12.73	5.0	0.612	0.251	0.095
1–3 (mild)	251	14.60 ± 13.30	11.0	0.406	0.375	0.219
4–6 (moderate)	320	19.18 ± 12.83	19.0	0.231	0.581	0.291
7–8 (severe)	208	21.88 ± 15.59	21.0	0.240	0.644	0.389

FIES Level, Food Insecurity Experience Scale total score categories; n, number of participants; BAI M ± SD, Beck Anxiety Inventory mean ± standard deviation; Median, BAI median score; *P* (BAI ≤ 7), conditional probability of minimal anxiety; *P* (BAI > 15), conditional probability of moderate-to-severe anxiety; *P* (BAI > 25), conditional probability of severe anxiety. Probabilities were computed as empirical proportions within each FIES category. All estimates were derived from the observed sample (*N* = 1,042).

Finally, the what-if analysis (Figure 3, Panel E) estimated the potential impact of hypothetical policy interventions. Reducing all participants to the Low EFINUB impact level was associated with a decrease in severe food insecurity from the sample baseline to 1.2% and in severe anxiety from the baseline to 17.6%, whereas achieving universal food security (FIES = 0) was associated with a reduction in severe anxiety to the level observed among food-secure individuals. These scenario-based projections highlighted the potential downstream benefits of early interventions targeting nutritional behavior changes during periods of food inflation.

Table 3 presents the empirical conditional probability distributions of anxiety severity across four food insecurity levels. A consistent monotonic relationship was observed between food insecurity severity and anxiety burden. Among fully food-secure individuals (FIES = 0), the mean BAI score was 9.63 ± 12.73 with a median of 5.0, and 61.2% of participants fell within the minimal anxiety range (BAI ≤ 7). As food insecurity deepened, these figures shifted substantially: in the severe food insecurity group (FIES = 7–8), the mean BAI score rose to 21.88 ± 15.59 with a median of 21.0, and the proportion of minimal anxiety decreased to 24.0%. Concurrently, the probability of moderate-to-severe anxiety (BAI > 15) increased from 25.1% in the food-secure group to 64.4% in the severely food-insecure group, while the probability of severe anxiety (BAI > 25) rose from 9.5% to 38.9%, an absolute increase of 29.4 percentage points (an approximately 4-fold relative increase). These findings provided empirical support for the FIES → BAI arc identified in the Bayesian Network structure and confirmed that food insecurity served as a strong probabilistic predictor of anxiety severity.

Regarding the role of income adequacy, model-based conditional probability queries indicated that *P*(*EFINUB*_*BT*_ = *High*)≈0.242 remained approximately constant across all income groups, which was consistent with the absence of a direct Income → EFINUB arc at the 0.85 bootstrap threshold (S = 0.76). However, empirical subgroup analysis revealed meaningful differences beneath this aggregate pattern. The vulnerable subgroup (*n* = 168), characterized by lower income adequacy, exhibited 32.7% High EFINUB prevalence, 26.8% severe food insecurity, and 30.4% severe anxiety, whereas the resilient subgroup (*n* = 129), characterized by higher income adequacy, demonstrated considerably lower rates at 19.4%, 18.6%, and 16.3%, respectively. This discrepancy between the model-based and empirical estimates suggested that income adequacy may have exerted its influence on outcomes through indirect pathways not captured by a single direct arc, or that the association fell just below the conservative threshold adopted for the primary network model.

To evaluate the sensitivity of the network structure to threshold selection, a supplementary analysis was conducted using a relaxed bootstrap strength threshold of 0.70 (Supplementary Figures S1, S2). Lowering the threshold from 0.85 to 0.70 resulted in the inclusion of additional arcs that had not met the more conservative criterion, while all arcs retained in the primary model remained present in the relaxed network. The distribution of bootstrap arc strength values across the full candidate set is presented in Supplementary Figure S2, Panel A, which illustrated a bimodal pattern with a concentration of arcs at the upper end of the strength spectrum and a secondary cluster near the lower boundary. Panel B displayed the number of arcs retained at seven candidate thresholds ranging from 0.50 to 0.95, confirming that the selected 0.85 threshold represented a balance between network parsimony and structural completeness. Importantly, the newly admitted arcs at the 0.70 threshold exhibited dashed lines in Supplementary Figure S1 to distinguish them from those retained from the primary model. These additional connections predominantly involved cross-domain links between demographic, lifestyle, and dietary habit variables, while the core pathway from EFINUB Food Consumption Impact through food insecurity to anxiety remained structurally invariant. This stability of the core dependency pathway across threshold specifications provided evidence that the primary findings were not artifacts of the particular threshold chosen and that the substantive conclusions regarding the mediating role of food insecurity between inflation-related behavioral changes and psychological distress were robust to analytical decisions in network construction.

A key advantage of Bayesian Networks over conventional regression models is their capacity to conduct counterfactual reasoning through conditional probability queries. By manipulating the values of specific nodes and propagating the resulting changes through the network, it becomes possible to estimate the potential downstream effects of hypothetical interventions on outcome variables. To leverage this capacity, a what-if analysis was performed using the fitted network, in which the values of two key variables—EFINUB Food Consumption Impact level and FIES Food Insecurity Experience—were systematically altered, and the resulting changes in the probabilities of severe food insecurity (FIES > 6) and severe anxiety (BAI > 25) were estimated relative to the observed sample baseline (Figure 3, Panel E; Table 4).

**Table 4:** Policy intervention what-if analysis: estimated probabilities of severe outcomes under counterfactual scenarios.

Scenario	*P* (FIES > 6)	Change	*P* (BAI > 25)	Change
Current sample	0.200	—	0.244	—
A: All EFINUB_BT_ = Low	0.012	−93.9%	0.176	−27.7%
B: All EFINUB_BT_ = Moderate	0.124	−37.7%	0.254	+4.3%
C: All FIES = 0	0.000	−100%	0.095	−61.0%
D: High → Moderate^*^	0.130	−72.5%	0.259	−10.9%
E: High → Low^*^	0.023	−95.2%	0.076	−73.8%

Scenarios A–B were estimated using model-based likelihood weighting; Scenarios C, D, and E were computed from empirical subgroup proportions. ^*^Change calculated relative to High group baseline [*P* (FIES > 6) = 0.474; *P* (BAI > 25) = 0.291].

Five counterfactual scenarios were constructed. Scenario A simulated a condition in which food inflation produced minimal disruption to dietary behaviors by assigning all participants to the Low Food Consumption Impact level. Scenario B represented a partial but incomplete behavioral adaptation by assigning all participants to the Moderate impact level. Scenario C modeled the hypothetical elimination of food insecurity by setting FIES = 0 for the entire sample, regardless of behavioral impact levels. The remaining scenarios focused specifically on the High-impact subgroup, which represented the most severely affected segment of the sample. Scenario D examined the effect of a partial intervention that shifted High-impact individuals to the Moderate category, whereas Scenario E examined the effect of a comprehensive intervention that shifted them to the Low category. Two estimation approaches were employed: Scenarios A and B were estimated using model-based likelihood weighting within the fitted Bayesian Network, which allowed the intervention effects to propagate through the full network structure, while Scenarios C, D, and E were computed from empirical subgroup proportions to provide complementary estimates grounded directly in the observed data.

The current sample baseline indicated that 20.0% of participants experienced severe food insecurity and 24.4% reported severe anxiety. Scenario A, in which all participants were assigned to the Low Food Consumption Impact group, produced a 93.9% reduction in severe food insecurity (from 20.0% to 1.2%) and a 27.7% reduction in severe anxiety (from 24.4% to 17.6%). Scenario B, which assigned all participants to the Moderate impact group, yielded a more modest reduction in severe food insecurity (12.4%, a 37.7% decrease) but produced a slight increase in severe anxiety (25.4%, a 4.3% increase). This divergent pattern suggested that moderate-level behavioral disruption was sufficient to sustain anxiety at near-baseline levels even when food insecurity was partially alleviated, indicating that the anxiety burden associated with food inflation may have been driven by the behavioral change experience itself rather than solely by its downstream nutritional consequences (Table 4).

Scenario C, which assumed universal food security (FIES = 0 for all participants), produced the largest reduction in severe anxiety among all scenarios examined (−61.0%, from 24.4% to 9.5%). This finding was particularly noteworthy as it demonstrated that direct food security interventions were more effective at reducing anxiety than indirect approaches targeting nutritional behavior changes. The magnitude of this effect indicated that a substantial proportion of the anxiety burden observed in the sample was attributable to the food insecurity experience itself, rather than to the behavioral disruptions that preceded it (Table 4).

Scenarios D and E evaluated the impact of shifting individuals from the High impact group to lower categories. Transitioning High-impact individuals to the Moderate group (Scenario D) reduced the within-group probability of severe food insecurity by 72.5% and severe anxiety by 10.9%. A more substantial shift to the Low group (Scenario E) achieved reductions of 95.2% and 73.8%, respectively. The contrast between these two scenarios highlighted a non-linear dose–response relationship: moving from High to Moderate yielded a substantial improvement in food insecurity but a relatively modest effect on anxiety, whereas moving from High to Low produced comprehensive improvements across both outcome domains. These results suggested that partial behavioral interventions may alleviate food insecurity without meaningfully addressing the accompanying psychological burden, and that more intensive interventions achieving Low-level behavioral impact would be required to generate clinically meaningful reductions in anxiety (Table 4).

To evaluate whether the observed network structure was driven by the gender composition of the sample, a supplementary analysis was conducted using a gender-balanced subsample (*n* = 564, 50% male). The full bootstrap stability procedure (1,000 resamples, threshold S ≥ 0.85) was repeated on this subsample, yielding 22 directed arcs. The structural similarity between the full-sample and balanced-sample networks was quantified using the Jaccard index, which yielded J = 0.808, indicating high structural agreement. Of the 25 arcs in the full-sample network, 21 were recovered in the balanced model. The four arcs not recovered were EFINUB Food Consumption Impact → Budget Pressure (S = 0.878 in the full sample), Employment → Gender (S = 0.984), Employment → Income Adequacy (S = 0.953), and Age Group → Chronic Disease (S = 0.938). Critically, the entire scale-relationship pathway — from EFINUB Food Consumption Impact through Perceived Food Insecurity, Panic Buying, Food Consumption Pattern, Food Shopping Behaviors, and Food Purchasing Motives to FIES Food Insecurity and ultimately BAI Anxiety — was preserved intact in the balanced model.

The balanced model recovered one additional arc not present in the full-sample network: Marital Status → Physical Activity. This connection, which fell just below the bootstrap threshold in the primary analysis, suggested that marital status may have exerted an influence on physical activity patterns that became more detectable in the gender-balanced subsample. The four arcs not recovered in the balanced model were predominantly demographic connections (Employment → Gender, Employment → Income Adequacy, Age Group → Chronic Disease) and one scale-relationship arc (EFINUB Food Consumption Impact → Budget Pressure, S = 0.878 in the full sample). The non-recovery of these arcs was attributable to reduced statistical power (*n* = 564 vs. 1,042) rather than structural instability, as all four had strength values below 0.98 in the primary model. Notably, the Budget Pressure node remained connected to the network through its parent Panic Buying (shared arc), indicating that only the direct influence from the EFINUB hub was attenuated, not the node's overall integration into the scale-relationship cluster. The high Jaccard index (J = 0.808) and the complete preservation of the core pathway from EFINUB Food Consumption Impact through FIES Food Insecurity to BAI Anxiety confirmed that the substantive conclusions of the primary analysis were robust to the gender composition of the sample (Supplementary Table S7).

In a further sensitivity analysis, retaining the EFINUB Food Consumption subscale as a continuous variable rather than categorizing it produced a structurally concordant network (Jaccard = 0.759), in which the core pathway from EFINUB Food Consumption through food insecurity to anxiety and the single-node (food insecurity) Markov blanket of anxiety were fully preserved (Supplementary Table S9).

## 4 Discussion

This study, conducted during a period of pronounced food inflation in Türkiye, examines the multivariate conditional dependency structure linking food inflation–related changes in dietary and consumer behaviors, food insecurity experiences, and anxiety using Bayesian Network analysis. The results indicate that behavioral changes driven by food inflation and food insecurity play a central and multidimensional role in shaping psychological health–related outcomes. Moreover, the results support that the Bayesian Network approach provides a robust methodological framework for uncovering these complex relationships and for evaluating potential policy and intervention scenarios. Through this integrative approach, the relationships among dietary and consumer behavior changes, food insecurity, and psychological outcomes are evaluated not only at a pairwise level but within directed and multivariate dependency structures.

The resulting network structure demonstrates that the impact of food inflation on dietary behavior is associated with food insecurity and anxiety through both direct and indirect pathways. This is consistent with previous research showing that Bayesian Networks are effective tools for modeling complex relationships between health behaviors and psychosocial outcomes (33–35). In addition, bootstrap-based structural validation and Markov blanket analyses indicate that the identified relationships are not only statistically significant but also structurally consistent and reproducible, supporting the usefulness of Bayesian Networks as a comprehensive framework for health research involving highly interconnected variables (36).

The most prominent result of the study is that food insecurity emerged as the sole direct conditional dependency of anxiety within the learned network. The Markov blanket of anxiety consisted exclusively of the food insecurity variable, indicating that, among the 22 remaining variables, food insecurity provided the minimum information required to explain anxiety in the model. This finding is consistent with the extensive literature showing strong associations between food insecurity and adverse psychological outcomes, including anxiety and depression (3, 37). During periods of economic uncertainty and inflation, food insecurity may become a particularly important psychosocial stressor (38). Accordingly, our results suggest that anxiety may be more closely linked to direct experiences of uncertainty and insufficiency in food access than to general sociodemographic risk factors alone.

The learned network also indicated that the effects of dietary and consumer behavior changes on anxiety operated primarily through food insecurity as an indirect pathway. This aligns with literature suggesting that psychosocial outcomes are often shaped not by abstract economic indicators *per se* but by individuals' concrete experiences of deprivation (3). A more detailed examination of the network revealed three distinct clusters. Although demographic variables formed a strong and internally consistent structure, they did not show direct connections—at the high confidence threshold of S ≥ 0.85—with scale variables related to food inflation and dietary behavior. This suggests that the behavioral effects of food inflation may operate through broadly similar mechanisms across age, education, and income groups. However, when the threshold was reduced to 0.70, bridging connections emerged, indicating that socioeconomic influences may operate indirectly through mechanisms such as budget pressure and food insecurity. This interpretation is consistent with evidence showing that lower income affects dietary quality and health outcomes primarily through food insecurity (39).

Descriptive results and group comparisons further supported the structural findings. EFINUB food consumption clusters identified via GMM differed significantly according to sex, education, income, and employment status, suggesting that demographic factors are not irrelevant but may exert their effects indirectly within the network structure. This interpretation is consistent with the capacity of Bayesian Networks to uncover mediation pathways and indirect interactions among variables (35).

Taken together, these observations provide a direct answer to the second research question, namely whether demographic characteristics play a direct or indirect role within the dependency structure. At the primary threshold (S ≥ 0.85), the demographic variables formed a coherent, internally connected cluster (organized around age group, employment, gender, marital status, and income adequacy) but showed no direct arcs to the food-inflation behavioral subscales, food insecurity, or anxiety. In other words, none of the demographic variables entered the Markov blanket of anxiety or of food insecurity, indicating that they exert no direct conditional dependency on the psychological outcome once the behavioral and food-insecurity variables are accounted for. However, their role is not negligible but rather indirect: when the threshold was relaxed to 0.70, bridging arcs emerged linking the demographic and socioeconomic variables (particularly income adequacy) to the scale cluster through budget pressure and food insecurity, and the GMM-derived food consumption impact groups differed significantly by gender, education, income, and employment. This pattern indicates that demographic and socioeconomic characteristics shape exposure to food-inflation-related behavioral disruption and food insecurity, which in turn relate to anxiety, rather than influencing anxiety directly. The demographic structure therefore operates upstream of, and indirectly through, the behavioral and food-insecurity pathway, consistent with the conditional-independence logic of the Bayesian Network and with evidence that socioeconomic disadvantage affects psychological outcomes largely via food insecurity (39).

Within the model, the variable representing changes in dietary behavior was positioned as a central node and was directly connected to perceived food insecurity, panic buying, budget pressure, and changes in food consumption. This configuration indicates that food inflation functions not only as an economic phenomenon but also as a multidimensional stressor shaping individual behaviors and decision-making processes. In network-based analyses, such central nodes are typically interpreted as critical control points that govern system behavior (35, 36). Bootstrap-based structural analysis further supported the robustness and directional consistency of these relationships. The directed chain from panic buying to budget pressure and subsequent purchasing behavior changes is consistent with behavioral economic models showing that consumer decisions are rapidly reorganized under conditions of uncertainty (40). Similarly, the strong pathway linking food insecurity to anxiety aligns with research indicating that economic stress affects behavioral and psychological outcomes through sequential and cumulative processes (40, 41).

Another notable result was the isolation of the dietary pattern cluster, comprising breakfast habits, meal skipping, and daily meal frequency. Considering the relatively young and highly educated sample, this pattern may suggest that meal-skipping behavior reflects lifestyle preferences rather than economic constraints. Irregular eating habits among university students and young adults have been widely documented (42). At the same time, diet quality is known to be sensitive to economic shocks (43). Therefore, food inflation may exert stronger effects on the type and nutritional content of foods consumed than on meal frequency. However, because detailed dietary composition was not measured, future studies incorporating nutrient intake and diet quality assessments are needed.

The substantial differentiation of FIES scores across EFINUB groups, with a large effect size, alongside the smaller but significant effect observed for anxiety, suggests that food inflation-related dietary disruption may primarily affect food insecurity, while psychological outcomes emerge as secondary consequences. The monotonic increase in both FIES and BAI scores across EFINUB groups supports a graded association between dietary behavior deterioration, food insecurity, and anxiety, consistent with previous literature on the psychological impact of food insecurity (3).

When the factorization of the network is considered alongside directed edges with high bootstrap strength, a dominant conditional dependency pathway emerges from EFINUB to perceived food insecurity, then to FIES and anxiety. This structure should not be interpreted as proof of causality, but rather as the most probable information flow within the learned network. The observed pattern suggests that the psychological effects of food inflation may be shaped largely through experiences of food insecurity rather than directly through macroeconomic indicators. This is consistent with prior studies reporting strong associations between food insecurity, anxiety, and psychological distress across diverse socioeconomic contexts (44–46).

The Markov blanket analysis further strengthens this interpretation. The fact that anxiety was conditionally dependent only on food insecurity implies that the effects of other variables, including demographic characteristics, lifestyle factors, and dietary behaviors, became statistically negligible once food insecurity was known. Food insecurity is not limited to nutritional deprivation; it also represents a powerful psychosocial stressor associated with chronic stress, anxiety, and depression (3, 37). This aligns with neuroscience evidence indicating that uncertainty and anticipation of negative outcomes are core mechanisms underlying anxiety (10), and food insecurity inherently involves persistent uncertainty about future food access. In the present study, this finding indicates that the direct experience of uncertainty and insufficiency in food access may be more relevant to anxiety than broad sociodemographic risk factors.

It is also noteworthy that perceived food insecurity and changes in food consumption emerged as parent variables of FIES. This indicates that food insecurity experiences are shaped not only by objective conditions of access but also by perceived insecurity and behavioral adaptation. This is consistent with research showing that food insecurity is a multidimensional construct linked to perceived stress, cost-of-living pressures, coping strategies, and changes in dietary behaviors (1, 47–49). Individuals experiencing food insecurity may shift toward lower-cost, nutrient-poor foods, modify meal content, and restructure purchasing behaviors, which may further intensify the experience of food insecurity (48). Thus, food insecurity cannot be explained solely by material deprivation; perceived insecurity and behavioral adaptation are integral components of this experience.

The connections among demographic variables showed high bootstrap strength but relatively low direction probabilities, suggesting directional uncertainty and potential bidirectionality. In contrast, the higher directional certainty observed from sex and marital status to BMI indicates that anthropometric outcomes were more consistently associated with specific demographic factors, in line with literature on sociodemographic determinants of body weight and lifestyle behaviors (50).

Conditional probability distributions and scenario analyses showed that the effects of food inflation-related dietary behavior changes on food insecurity and anxiety followed a non-linear but strongly graded pattern. The marked rise in severe food insecurity from low to high EFINUB levels—an absolute increase of 47.0 percentage points (from 1.2% to 48.2%)—suggests that the impact of food inflation reflects an accelerating accumulation of risk rather than a marginal change. We interpret this primarily in absolute terms; while the corresponding relative change is large (approximately 40-fold), the low baseline prevalence warrants caution in emphasizing the fold-change figure. This is consistent with studies showing that economic shocks can affect food insecurity nonlinearly once certain thresholds are exceeded (43, 51). Global analyses similarly indicate that food price increases disproportionately amplify food insecurity risk, particularly among vulnerable populations (1). Consistent with this, macro-level causal analyses spanning multiple countries have shown that rising food prices and income inequality jointly drive population food insecurity, which in turn elevates downstream health burdens (52). At the country level, time-series causality evidence from Türkiye further links nutrition-related risk factors to rising health expenditure (53), underscoring that the individual-level food-insecurity pathway identified here is embedded within broader macroeconomic dynamics linking food price inflation to public health costs.

Although the association between EFINUB and anxiety was more limited, it still showed an approximately 2-fold increase, suggesting that dietary behavior changes may generate psychological burden, especially when they are accompanied by food insecurity. The monotonic increase in anxiety scores across FIES levels further confirms that the FIES–anxiety relationship is evident not only in the network structure but also in empirical distributions. Similar patterns have been reported in large-scale studies showing higher anxiety and psychological distress among individuals experiencing food insecurity (3, 45), particularly during periods of crisis and economic strain (38).

The what-if scenario analyses clarify the policy relevance of these findings. Shifting all individuals to a low EFINUB level was associated with large reductions in severe food insecurity and smaller reductions in severe anxiety, whereas the full food security scenario produced the most marked decrease in anxiety. This suggests that interventions focusing only on dietary behavior may be insufficient, whereas structural policies that directly ensure food access—such as food subsidies, social support programs, and food banks—may have stronger implications for psychological health. Previous research similarly shows that food assistance programs can reduce both food insecurity and psychological stress (41, 47).

Nevertheless, these scenario analyses should not be interpreted as causal inference. They represent Bayesian Network–based probabilistic projections grounded in the observed conditional dependency structure and may not fully reflect the real-world impact of interventions. The presence of unobserved confounders and the cross-sectional design require cautious interpretation within a limited inferential framework (22). Despite these limitations, the analyses provide useful insight into potential intervention points in the context of food inflation.

Discrepancies between model-based and empirical results regarding income adequacy also provide important methodological insight. Although the Bayesian Network did not identify a direct edge between income level and dietary behavior, subgroup analyses showed that lower-income individuals had higher dietary behavior impact, severe food insecurity, and anxiety. This suggests that income-related effects may operate indirectly through intermediary mechanisms, consistent with prior research showing that socioeconomic disadvantage affects health outcomes through food insecurity and related pathways (47, 54).

Sensitivity analyses further supported the robustness of the findings. Although lowering the bootstrap threshold from 0.85 to 0.70 introduced additional edges, the primary pathway from dietary behavior impact to food insecurity and anxiety remained unchanged. This aligns with methodological literature showing that Bayesian Networks can preserve core dependency structures across varying threshold specifications (11). Similarly, analyses in a sex-balanced subsample showed high structural similarity, with complete preservation of the main pathway. The missing edges mostly involved demographic variables or weaker associations, suggesting reduced statistical power rather than structural instability.

Overall, the results demonstrate that food inflation-related dietary behavior changes are embedded within multilayered relationships involving food insecurity and anxiety. The Bayesian Network approach not only identified existing conditional dependencies but also enabled system-level evaluation of potential intervention scenarios. Although these findings should not be interpreted causally, they indicate that food insecurity occupies a central position in the system. Therefore, mitigating the psychological impacts of food inflation may require multidimensional strategies that address not only income and consumer behavior but also direct food access, price stability, and food security.

This study has several important strengths. The use of a Bayesian Network approach enables the modeling of complex and multidimensional relationships among dietary behavior changes, food insecurity, and anxiety. Unlike traditional regression methods, this approach allows for the analysis of conditional dependencies and the evaluation of intervention effects through counterfactual scenarios. This contributes to interpreting the results not only at a relational level but also in a manner that is meaningful for policy applications. The combined use of likelihood-weighted sampling and empirical probability estimation allowed for the comparison of model-based and data-driven results, thereby enhancing the robustness of the findings. The implementation of “what-if” scenario analyses further extended the study beyond a purely descriptive framework, enabling the evaluation of potential intervention effects. In addition, structural stability analyses conducted on a sex-balanced subsample and bootstrap procedures support the reliability of the derived network structure. The 1,000-bootstrap iterations and multiple sensitivity analyses demonstrate that the findings are structurally consistent. The high structural similarity coefficient indicates that the model is only minimally sensitive to sample composition and that core relationships are consistently preserved. Finally, conducting the study in the context of food inflation—a timely and policy-relevant issue—enhances both the external validity and practical significance of the results.

This study also has several limitations. First, due to its cross-sectional design, causal inferences are limited. The relationships identified cannot be interpreted causally, and directed connections in Bayesian Networks reflect statistical dependencies rather than causal mechanisms. Although Bayesian Networks are capable of modeling directed dependencies, longitudinal or experimental designs are required to establish causality with greater certainty. Therefore, the relationships identified in this study should be interpreted as conditional dependencies. Second, the sample, recruited online through convenience and snowball sampling, was concentrated among younger adults (57.0% aged 18–24) and women (72.9%) and was predominantly university-educated, which limits the generalizability of the prevalence estimates to the broader Turkish population. The sample composition was formally compared against national population parameters (Supplementary Table S5), which transparently documents these deviations; accordingly, the findings are best interpreted as describing the conditional dependency structure among these constructs rather than as nationally representative population estimates. The use of self-reported data may also introduce measurement errors such as recall bias and social desirability bias. In addition, the online data collection method may have excluded individuals with limited internet access, thereby constraining sample representativeness. Third, the presence of unmeasured confounders not included in the model may have influenced the observed relationships. Although Bayesian Networks model dependencies among observed variables, the influence of latent variables cannot be fully eliminated. Furthermore, reduced sample sizes in certain subgroup analyses may have decreased statistical power, potentially limiting the detection of some relationships. The use of categorical thresholds for variables facilitates interpretability but may lead to the loss of nuanced information in continuous distributions, particularly at extreme and intermediate values. However, a sensitivity analysis in which the EFINUB Food Consumption subscale was modeled as a continuous variable yielded a highly concordant network structure (Jaccard = 0.759) with an identical single-node (food insecurity) Markov blanket for anxiety, indicating that this information loss did not materially affect the principal findings (Supplementary Table S9). Finally, although the FIES items satisfied the assumptions of the Rasch measurement model in this sample (Supplementary Section S10), the sample-specific scale was not equated to the FAO global reference metric; consequently, the food-insecurity categories reported here reflect raw-score cutpoints rather than internationally comparable, calibrated prevalence estimates of moderate-or-severe and severe food insecurity, and cross-national prevalence comparisons should be made with caution. As food insecurity is a central variable in this study, future applications would benefit from the full FAO calibration and equating procedure to enable direct comparability with SDG indicator 2.1.2 estimates.

Future research is recommended to employ longitudinal designs in order to better elucidate causal relationships. Repeated measurements over time would contribute to a clearer understanding of the dynamic relationships among dietary behavior changes, food insecurity, and anxiety. In addition, incorporating objective dietary assessment methods (e.g., food consumption records, biomarkers) alongside self-reported data may improve measurement accuracy. The scope of the model could be expanded by including additional psychosocial and environmental variables such as coping mechanisms, social support, and economic vulnerability. Methodologically, comparing Bayesian Network analysis with other causal inference approaches such as structural equation modeling (SEM) or directed acyclic graphs (DAG) would be valuable for assessing model robustness. Furthermore, studies incorporating larger and more diverse samples across different socioeconomic and cultural contexts would enhance the generalizability of the results. Finally, testing real-world interventions based on the scenario analyses proposed in this study would facilitate the translation of the results into policy and public health practice.

## 5 Conclusion

This study showed that food insecurity occupies a central position in the network linking food inflation-related dietary behavior changes with anxiety among adults in Türkiye. In the learned Bayesian Network, anxiety was conditionally dependent primarily on food insecurity, suggesting that the psychological burden of food inflation may operate through concrete experiences of constrained food access rather than demographic characteristics alone. Scenario-based probability analyses further indicated that direct improvements in food security may be more strongly associated with reductions in anxiety symptoms than partial behavioral changes. These findings highlight food insecurity as both a nutritional and mental health concern and support the need for integrated policies that improve food access, dietary resilience, and psychological wellbeing during inflationary periods.

## Data Availability

The datasets presented in this article are not readily available because of ethical and privacy restrictions related to participant data, in accordance with the approval of the ethics committee; the data may be made available by the corresponding author upon reasonable request and with appropriate ethical approval. The R code used for all analyses is publicly available in a Zenodo repository (https://doi.org/10.5281/zenodo.20465160). Requests to access the datasets should be directed to salimyilmaz142@gmail.com.

## References

[1] FAO IFAD UNICEF WFP and WHO. The State of Food Security and Nutrition in the World 2023. Urbanization, Agrifood Systems Transformation and Healthy Diets Across the Rural–Urban Continuum. Rome, FAO (2023).

[2] FAO. State of Food Security and Nutrition in the World 2025: Price Inflation and Food Security (2025). Available online at: https://openknowledge.fao.org/items/4b1f7d26-267d-4a81-aed4-4f9de4d93f85 (Accessed May 02, 2026).

[3] Pourmotabbed A Moradi S Babaei A Ghavami A Mohammadi H Jalili C . Food insecurity and mental health: a systematic review and meta-analysis. Public Health Nutr. (2020) 23:1778–90. doi: 10.1017/S136898001900435X 32174292 PMC10200655

[4] Myers CA. Food insecurity and psychological distress: a review of the recent literature. Curr Nutr Rep. (2020) 9:107–18. doi: 10.1007/s13668-020-00309-1 32240534 PMC7282962

[5] Bateson M Chevallier C Johnson EA Johnson MT Pickett KE Nettle D . Does food insecurity cause anxiety and depression? Evidence from the changing cost of living study. PLoS Mental Health. (2025) 2:e0000320. doi: 10.1371/journal.pmen.0000320 41661973 PMC12798610

[6] Jackson K Kelemen Z Nagy Á. Inflation, food insecurity, and mental health: Generation Z's burden in emerging Europe. Human Soc Sci Commun. (2025) 12:1–12. doi: 10.1057/s41599-025-05858-w

[7] Kilinç GE Vergi Y Keser A. Validation and reliability of the Turkish version of the food insecurity experience scale (FIES) among adults. J Health Popul Nutr. (2025) 44:264. doi: 10.1186/s41043-025-00873-8 40702520 PMC12285091

[8] Turkish Statistical Institute (Turkstat). Consumer Price Index, December 2025 (Bulletin No. 58294) (2026). Available online at: https://veriportali.tuik.gov.tr/tr/press/58294 (Accessed May 02, 2026).

[9] Haydaroglu M Kopuz TNY Güneş FE. Exploring the associations between food security and multidimensional well-being under economic uncertainty: a cross-sectional study in Türkiye. BMC Public Health. (2025) 25:3826. doi: 10.1186/s12889-025-25152-3 41204230 PMC12595700

[10] Grupe DW Nitschke JB. Uncertainty and anticipation in anxiety: an integrated neurobiological and psychological perspective. Nat Rev Neurosci. (2013) 14:488–501. doi: 10.1038/nrn3524 23783199 PMC4276319

[11] Scutari M Denis J-B. Bayesian Networks: With Examples in R (2nd ed.). New York, NY: CRC Press (2021).

[12] Eşer Durmaz S Keser A Ergenç C. A multidimensional scale for evaluating food inflation's impact on nutritional behavior. BMC Public Health. (2025) 25:2298. doi: 10.1186/s12889-025-23553-y 40610920 PMC12224678

[13] Haydaroglu M Bilgiç P. Validation of the food inflation impact on consumer behavior scale: a comparative measurement instrument with focus on food security. Int J Food Sci Nutr. (2024) 16:1–12. doi: 10.1080/09637486.2024.2379819 39014966

[14] Ballard TJ Kepple AW Cafiero C. The Food Insecurity Experience Scale: Development of a Global Standard for Monitoring Hunger Worldwide. Rome: FAO (2013).

[15] Cafiero C Viviani S Nord M. Food security measurement in a global context: the food insecurity experience scale. Measurement. (2018) 116:146–52. doi: 10.1016/j.measurement.2017.10.065

[16] Beck AT Epstein N Brown G Steer RA. An inventory for measuring clinical anxiety: psychometric properties. J Consult Clin Psychol. (1988) 56:893–7. doi: 10.1037/0022-006X.56.6.893 3204199

[17] Ulusoy M Sahin NH Erkmen H. Turkish version of the beck anxiety inventory: psychometric properties. J Cogn Psychother. (1998) 12:163–72.

[18] Celeux G Soromenho G. An entropy criterion for assessing the number of clusters in a mixture model. J Classif . (1996) 13:195–212. doi: 10.1007/BF01246098

[19] Curran PJ West SG Finch JF. The robustness of test statistics to nonnormality and specification error in confirmatory factor analysis. Psychol Methods. (1996) 1:16–29. doi: 10.1037/1082-989X.1.1.16

[20] Kline RB. Principles and Practice of Structural Equation Modeling (4th ed.). New York, NY: Guilford Press (2016).

[21] Scutari M Nagarajan R. Identifying significant edges in graphical models of molecular networks. Artif Intell Med. (2013) 57:207–17. doi: 10.1016/j.artmed.2012.12.006 23395009 PMC4070079

[22] Pearl J. Causality: Models, Reasoning, and Inference (2nd ed.). Cambridge: Cambridge University Press (2009).

[23] R Core Team. R: A Language and Environment for Statistical Computing. Vienna: R Foundation for Statistical Computing (2025).

[24] Revelle W. psych: Procedures for Psychological, Psychometric, and Personality Research. Evanston, IL: Northwestern University (2025).

[25] Cafiero C Viviani S Nord M. RM.weights: Weighted Rasch Modeling and Extensions using Conditional Maximum Likelihood [R package version 2.0]. Vienna: CRAN (2018).

[26] Food and Agriculture Organization of the United Nations. Using the FIES App: A Simple Tool for the Analysis of Food Insecurity Experience Scale data. Rome: FAO (2020).

[27] Scrucca L Fraley C Murphy TB Raftery AE. Model-Based Clustering, Classification, and Density Estimation Using mclust in R. Boca Raton, FL: Chapman Hall/CRC (2023).

[28] Scutari M. Learning Bayesian networks with the bnlearn R package. J Statist Softw. (2010) 35:1–22. doi: 10.18637/jss.v035.i03

[29] Csárdi G Nepusz T. The igraph Software Package for Complex Network Research (2006). Available online at: https://mathematics.foi.hr/Rprojekti/grafovi/PDF/The_Igraph_Software_Package_for_Complex_Network_Re.pdf (Accessed May 02, 2026).

[30] Csárdi G Nepusz T Traag V Horvát S Zanini F Noom D . igraph: Network Analysis and Visualization in R. R package version 2.2.1 (2026). Available online at: https://CRAN.R-project.org/package=igraph (Accessed May 02, 2026).

[31] Pedersen TL. ggraph: An Implementation of Grammar of Graphics for Graphs and Networks. R package version 2.2.2. (2025). Available online at: https://CRAN.R-project.org/package=ggraph (Accessed May 02, 2026).

[32] Wickham H. ggplot2: Elegant Graphics for Data Analysis. New York, NY: Springer-Verlag (2016).

[33] Calderon A Baik SY Ng MH Fitzsimmons-Craft EE Eisenberg D Wilfley DE . Machine learning and Bayesian network analyses identifies associations with insomnia in a national sample of 31,285 treatment-seeking college students. BMC Psychiatry. (2024) 24:656. doi: 10.1186/s12888-024-06074-7 39367432 PMC11452987

[34] Delgadillo J Budimir S Barkham M Humer E Pieh C Probst T . A Bayesian network analysis of psychosocial risk and protective factors for suicidal ideation. Front Public Health. (2023) 11:1010264. doi: 10.3389/fpubh.2023.1010264 36935710 PMC10014716

[35] Kyrimi E McLachlan S Dube K Neves MR Fahmi A Fenton N . A comprehensive scoping review of Bayesian networks in healthcare: past, present and future. Artif Intell Med. (2021) 117:102108. doi: 10.1016/j.artmed.2021.102108 34127238

[36] Friedman N Goldszmidt M Wyner A. Data analysis with Bayesian networks: a bootstrap approach. *arXiv* [Preprint]. arXiv:1301, 6695 (2013). Available online at: https://arxiv.org/abs/1301.6695 (Accessed January 13, 2013).

[37] Arenas DJ Thomas A Wang JC DeLisser HM. A systematic review and meta-analysis of depression, anxiety, and sleep disorders in US adults with food insecurity. J Gen Intern Med. (2019) 34:2874–82. doi: 10.1007/s11606-019-05202-4 31385212 PMC6854208

[38] Wolfson JA Leung CW. Food insecurity and COVID-19: disparities in early effects. Am J Public Health. (2020) 110:1763–5. doi: 10.2105/AJPH.2020.305953 32970451 PMC7662000

[39] Hanson KL Connor LM. Food insecurity and dietary quality in US adults and children. Am J Clin Nutr. (2014) 100:684–92. doi: 10.3945/ajcn.114.084525 24944059

[40] Sheth J. Impact of COVID-19 on consumer behavior: will the old habits return or die? J Business Res. (2020) 117:280–3. doi: 10.1016/j.jbusres.2020.05.059 32536735 PMC7269931

[41] Loopstra R. Interventions to address household food insecurity in high-income countries. Proc Nutr Soc. (2018) 77:270–81. doi: 10.1017/S002966511800006X 29580316

[42] Sogari G Velez-Argumedo C Gómez MI Mora C. College students and eating habits: a study using an ecological model for healthy behavior. Nutrients. (2018) 10:1823. doi: 10.3390/nu10121823 30477101 PMC6315356

[43] Headey DD Alderman HH. The relative caloric prices of healthy and unhealthy foods differ systematically across income levels. J Nutr. (2019) 149:2020–33. doi: 10.1093/jn/nxz158 31332436 PMC6825829

[44] Jones AD. Food insecurity and mental health status: a global analysis of 149 countries. Am J Prev Med. (2017) 53:264–273. doi: 10.1016/j.amepre.2017.04.008 28457747

[45] Weaver LJ Hadley C. Moving beyond hunger and nutrition: a systematic review of the evidence linking food insecurity and mental health in developing countries. Ecol Food Nutr. (2009) 48:263–84. doi: 10.1080/03670240903001167 21883069

[46] Loopstra R. Vulnerability to Food Insecurity Since the COVID-19 Lockdown. London: The Food Foundation (2020).

[47] Gundersen C Ziliak JP. Food insecurity and health outcomes. Health Affairs. (2015) 34:1830–9. doi: 10.1377/hlthaff.2015.0645 26526240

[48] Pooler JA Hartline-Grafton H DeBor M Sudore RL Seligman HK. Food insecurity: a key social determinant of health for older adults. J Am Geriatr Soc. (2019) 67:421–4. doi: 10.1111/jgs.15736 30586154 PMC6816803

[49] Nikolaus CJ An R Ellison B Nickols-Richardson SM. Food insecurity among college students in the United States: a scoping review. Adv Nutr (2020) 11:327–48. doi: 10.1093/advances/nmz111 31644787 PMC7442331

[50] Hruby A Hu FB. The epidemiology of obesity: a big picture. Pharmacoeconomics. (2015) 33:673–89. doi: 10.1007/s40273-014-0243-x 25471927 PMC4859313

[51] Laborde D Martin W Swinnen J Vos R. COVID-19 risks to global food security. Science. (2020) 369:500–2. doi: 10.1126/science.abc4765 32732407

[52] Günal AM Cantürk S Yilmaz S Boz C Karabay D. Examining the interconnections among income, food prices, food insecurity, and health expenditure: a multicausality approach. BMC Public Health. (2025) 25:2778. doi: 10.1186/s12889-025-24153-6 40813665 PMC12351902

[53] Yilmaz S Boz C Eren FA Günal AM. Unraveling the causal relationship between non-communicable diseases, obesity, and health expenditure: Insights from the Toda–Yamamoto approach. Healthcare. (2025) 13:1. doi: 10.3390/healthcare13010001 39791608 PMC11720058

[54] Tarasuk V Li T Fafard St-Germain AA. Household Food Insecurity in Canada 2021 (2022). Available online at: https://homelesshub.ca/wp-content/uploads/2024/04/Household-Food-Insecurity-in-Canada-2021-PROOF63.pdf (Accessed May 02, 2026).

[55] Nord M. Introduction to Item Response Theory Applied to Food Security Measurement: Basic Concepts, Parameters and Statistics. Rome: FAO (2014).

