# Supplementary material for: Food inflation, nutrition behavior, food insecurity, and anxiety: a Bayesian network analysis among Turkish adults
Source: Front Nutr. 2026 Jul 10;13:1865375. doi: 10.3389/fnut.2026.1865375 (PMC13395739; doi:10.3389/fnut.2026.1865375)
Supplement: Supplementary file 1 [file Table_1.docx]

**Supplementary Material**

*Food Inflation, Nutritional Behavior, Food Insecurity, and Anxiety: A Bayesian Network Analysis Among Turkish Adults*

This Supplementary Material provides detailed documentation of the analytical procedures, variable specifications, and sensitivity analyses supporting the findings presented in the main text. Section S1 presents the complete variable definitions and coding schemes for all 23 nodes in the Bayesian Network model. Section S2 documents the participant exclusion flow from initial responses to the final analytic sample. Section S3 reports the Gaussian Mixture Model sensitivity analysis used to derive the data-driven categorization of the EFINUB Food Consumption subscale. Section S4 assesses the representativeness of the study sample relative to national population parameters. Section S5 examines the sensitivity of the network structure to the bootstrap strength threshold selection, including supplementary network visualizations at the relaxed threshold of 0.70. Section S6 presents the gender-balanced sensitivity analysis, comparing the arc structures of the full-sample and balanced-sample networks. Section S7 documents the computational environment and reproducibility parameters. All table and figure numbering follows the S-prefix convention to distinguish supplementary content from the main text.

# S1. Variable Definitions and Coding Scheme

The Bayesian Network model incorporated 23 variables (nodes): 15 categorical and 8 continuous. Table S1 presents the complete variable list, including R variable names used in the analysis code, English labels used throughout the manuscript, domain classification, variable types, and detailed coding schemes. The variable ordering follows the domain structure used in the network visualization: demographic variables first, followed by anthropometric, lifestyle, dietary habits, EFINUB subscales, IFI-ConB subscales, and outcome variables.

**Table S1.** Complete variable list with definitions, types, and coding schemes for the Bayesian Network model (23 nodes).

| **Variable (R name)** | **English Label** | **Domain** | **Type** | **Coding / Range** |
| --- | --- | --- | --- | --- |
| cinsiyet | Gender | Demographic | Cat | Male; Female |
| yas_grubu | Age Group | Demographic | Cat | 18–24; 25–34; 35–44; 45+ |
| gelir_kat | Income Adequacy | Demographic | Cat | Below expenses; Equal to expenses; Above expenses |
| egitim_kat | Education | Demographic | Cat | Below high school; High school; University and above |
| kronik_var | Chronic Disease | Demographic | Cat | Yes (diagnosed); No |
| medeni | Marital Status | Demographic | Cat | Single; Married |
| calisma | Employment | Demographic | Cat | Employed; Unemployed |
| BKI_kat | BMI Category | Anthropometric | Cat | Normal/Underweight (<25); Overweight (25–29.9); Obese (≥30) |
| fiziksel_aktivite | Physical Activity | Lifestyle | Cat | Yes (regular); No |
| sigara | Smoking | Lifestyle | Cat | Yes (current); No |
| alkol | Alcohol | Lifestyle | Cat | Yes (current); No |
| kahvalti_kat | Breakfast Habit | Dietary Habits | Cat | Every morning; Sometimes; Rarely/Never |
| ogun_sayisi_kat | Meals per Day | Dietary Habits | Cat | 1; 2; 3; 4+ |
| ogun_atlama | Meal Skipping | Dietary Habits | Cat | No; Sometimes; Yes |
| BDE_BT_gmm | EFINUB Food Consumption Impact | EFINUB Subscales | Cat* | Low; Moderate; High (GMM-derived from items 1–22) |
| BDE_gida_guvencesizligi | EFINUB Perceived Food Insecurity | EFINUB Subscales | Cont | Items 23–27; range 5–25 |
| BDE_panik_alisveris | EFINUB Panic Buying | EFINUB Subscales | Cont | Items 28–33; range 6–30 |
| BDE_butce | EFINUB Budget Pressure | EFINUB Subscales | Cont | Items 34–36; range 3–15 |
| TDE_gida_tuketim | IFI-ConB Food Consumption Pattern | IFI-ConB Subscales | Cont | Items 1–6; range 0–18 |
| TDE_gida_alisveris | IFI-ConB Food Shopping Behaviors | IFI-ConB Subscales | Cont | Items 7–12; range 0–18 |
| TDE_satin_alma_motiv | IFI-ConB Food Purchasing Motives | IFI-ConB Subscales | Cont | Items 13–17; range 0–15 |
| FIES_toplam | FIES Food Insecurity Experience | Outcome | Cont | 8 items; range 0–8 (dichotomous scoring) |
| BAI_toplam | BAI Anxiety | Outcome | Cont | 21 items; range 0–63 (4-point Likert) |

*Cat = categorical; Cont = continuous; Cat* = originally continuous, categorized via Gaussian Mixture Model (see Section S3). EFINUB = The Effect of Food Inflation on Nutrition Behavior; IFI-ConB = The Impact of Food Inflation on Consumer Behavior; FIES = Food Insecurity Experience Scale; BAI = Beck Anxiety Inventory. BMI = body mass index, calculated from self-reported height and weight.*

# S2. Participant Exclusion Flow

The initial dataset comprised 1,148 responses collected via Google Forms between November and December 2025. A sequential exclusion procedure was applied to ensure data quality and eligibility.

**Table S2.** Sequential participant exclusion summary.

| **Step** | **Excluded (n)** | **Reason** | **Remaining (n)** | **Attrition (%)** |
| --- | --- | --- | --- | --- |
| Initial responses | — | — | 1,148 | — |
| Step 1: Consent screening | 30 | Non-consent | 1,118 | 2.6% |
| Step 2: Eligibility screening | 44 | Missing or ineligible age (< 18 years) | 1,074 | 6.4% |
| Step 3: Residency screening | 23 | Non-Türkiye | 1,051 | 8.4% |
| Step 4: Complete case (BN model) | 9 | Missing BMI | 1,042 | 9.2% |

*Step 4 removed observations with missing BMI values to ensure complete data across all 23 nodes. The final analytic sample of 1,042 complete cases was used for all analyses, including both descriptive statistics (Table 1) and the Bayesian Network model. Cumulative attrition calculated relative to the initial 1,148 responses.*

Table S2 summarizes the exclusion steps, the number of cases removed at each stage, the cumulative sample size, and the cumulative attrition rate.

# S3. Gaussian Mixture Model Sensitivity Analysis

To categorize the EFINUB Food Consumption subscale (items 1–22) into behaviorally meaningful subgroups, Gaussian Mixture Models were fitted using the mclust package in R (Scrucca et al., 2023). Models with 1 to 6 components were evaluated under both equal-variance (E) and variable-variance (V) structures. Table S3 presents the model comparison results.

**Table S3.** Gaussian Mixture Model comparison for the EFINUB Food Consumption subscale: model fit indices across 1–6 component solutions.

| **Variance** | **K** | **BIC** | **ICL** | **Entropy** | **Note** |
| --- | --- | --- | --- | --- | --- |
| E | 1 | −9743.79 | −9743.79 | — |  |
| V | 1 | −9743.79 | −9743.79 | — |  |
| E | 2 | −9688.44 | −9966.36 | 0.6219 |  |
| V | 2 | −9525.64 | −9571.79 | 0.9217 | *Best ICL* |
| E | 3 | −9617.52 | −9868.93 | 0.7778 |  |
| **V*** | **3*** | **−9514.63*** | **−9816.48*** | **0.7344*** | **Best BIC*** |
| E | 4 | −9616.18 | −9947.72 | 0.7641 |  |
| V | 4 | NA | NA | NA | *Not fitted* |
| E | 5 | −9518.81 | −9722.96 | 0.8787 |  |
| V | 5 | NA | NA | NA | *Not fitted* |
| E | 6 | −9525.29 | −9819.22 | 0.8424 |  |
| V | 6 | NA | NA | NA | *Not fitted* |

*K = number of components; E = equal-variance; V = variable-variance; BIC = Bayesian Information Criterion; ICL = Integrated Complete-data Likelihood. * = selected model (best BIC among interpretable solutions). NA = model did not converge under the variable-variance structure. Entropy = relative entropy of the classification (range 0–1; higher values indicate better classification certainty; not applicable for single-component models). The V,3 model achieved the best BIC (−9514.63) while the V,2 model achieved the best ICL (−9571.79); the V,3 model was selected based on BIC optimization and substantive interpretability of the three-group solution.*

The selected 3-component variable-variance model identified three distinct subgroups with the characteristics presented in Table S4. The relative entropy of 0.734 exceeded the conventional 0.60 threshold recommended for mixture model applications (Celeux & Soromenho, 1996), indicating acceptable classification certainty. Although higher-component models (4–6 components) achieved marginally better fit statistics, the additional components did not correspond to substantively interpretable subgroups and were driven by minor distributional irregularities rather than meaningful behavioral distinctions.

**Table S4.** GMM classification characteristics for the 3-component variable-variance model.

| **Group** | **n** | **%** | **Score Range** | **M** | **SD** | **Mean Post. Prob.** | **Entropy Contrib.** |
| --- | --- | --- | --- | --- | --- | --- | --- |
| Low | 131 | 12.6% | 22–26 | 23.4 | 1.5 | 0.882 | 0.040 |
| Moderate | 660 | 63.3% | 27–80 | 55.9 | 14.9 | 0.910 | 0.123 |
| High | 251 | 24.1% | 81–110 | 94.0 | 9.0 | 0.799 | 0.099 |
| **Total** | **1,042** | **100%** | **22–110** | — | — | **0.880** | **0.734** |

*M = mean; SD = standard deviation; Mean Post. Prob. = average posterior classification probability for members assigned to each group; Entropy Contrib. = each group's contribution to total classification uncertainty. Entropy (Total row) = overall relative entropy of the classification. M and SD reflect the empirical descriptive statistics of observations assigned to each group.*

GMM was also evaluated for the remaining six continuous subscales (EFINUB Food Insecurity, EFINUB Panic Buying, EFINUB Budget, IFI-ConB Food Consumption Pattern, IFI-ConB Food Shopping Behaviors, IFI-ConB Food Purchasing Motives). However, none of these subscales yielded tractable solutions: all produced optimal models with 5–6 components, attributable to narrow score range artifacts and ceiling/floor effects rather than substantively meaningful subgroup structures. These subscales were therefore retained as continuous variables in the Bayesian Network model, where they were parameterized using the Conditional Gaussian framework described in Section 2.5.2 of the main text.

# S4. Sample Representativeness Assessment

Because a non-probability sampling strategy combining convenience and snowball techniques was employed, the composition of the study sample was compared against available national population parameters. Table S5 presents this comparison.

**Table S5.** Comparison of study sample composition (n = 1,042) with national population parameters.

| **Variable** | **Sample (%)** | **National (%)** | **Source** | **Difference** |
| --- | --- | --- | --- | --- |
| Female | 72.9% | ~50% | TURKSTAT 2024 | Over-represented |
| Age 18–24 | 57.0% | ~15–18% | TURKSTAT 2024 | Over-represented |
| University+ | 80.6% | ~23–25% | TURKSTAT 2024 | Over-represented |
| Single | 73.6% | ~30–35% | TURKSTAT 2024 | Over-represented |
| Unemployed | 57.8% | — | TURKSTAT 2024 | — |
| Current smoker | 35.9% | ~28–32% | TURKSTAT 2022 | Comparable |
| Alcohol use | 10.1% | ~12–15% | TURKSTAT 2022 | Comparable |
| Obese (BMI ≥30) | 11.1% | ~21–23% | TURKSTAT 2022 | Under-represented |
| Chronic disease | 11.4% | — | — | — |

*National estimates sourced from the Turkish Statistical Institute (TURKSTAT) Address-Based Population Registration System (2024) and Turkish Health Survey (2022). Approximate ranges (∼) reflect variability across reporting years and age-group definitions. — = national comparison not available or not directly comparable.*

The study sample showed notable over-representation of females, younger adults, university-educated individuals, and single/unmarried respondents, consistent with the online convenience sampling method. Several considerations mitigate the impact of these distributional differences on the Bayesian Network findings. First, BN structure learning algorithms identify conditional dependency relationships rather than marginal prevalence estimates, and simulation studies have demonstrated that BN structure recovery is relatively robust to non-representative sampling when the underlying conditional independence relationships are stable across subpopulations (Scutari & Denis, 2021). Second, the gender-balanced sensitivity analysis (Section S6) confirmed that the core network structure was preserved when the gender imbalance was corrected. Third, the bootstrap stability assessment (1,000 resamples) provided an internal validation mechanism independent of external representativeness. Nonetheless, the marginal probability estimates should be interpreted with caution and may not reflect the true population prevalence.

# S5. Bootstrap Threshold Sensitivity Analysis

The primary Bayesian Network was constructed using a bootstrap strength threshold of S ≥ 0.85, retaining 25 directed arcs among 23 nodes. To assess the sensitivity of the network structure to this threshold choice, a supplementary analysis was conducted at a relaxed threshold of S ≥ 0.70. Table S6 presents the complete arc list, with the 25 retained arcs from the primary model and the four additional arcs admitted at the lower threshold.

**Table S6.** Bootstrap arc strength and direction probability at the relaxed threshold (S ≥ 0.70).

| **From** | **To** | **Strength** | **Direction** | **Status** |
| --- | --- | --- | --- | --- |
| EFINUB Food Consumption Impact | Perceived Food Insecurity | 1.000 | 1.000 | Retained |
| EFINUB Food Consumption Impact | Panic Buying | 0.976 | 1.000 | Retained |
| EFINUB Food Consumption Impact | Budget Pressure | 0.878 | 1.000 | Retained |
| EFINUB Food Consumption Impact | Food Consumption Pattern | 0.988 | 1.000 | Retained |
| Perceived Food Insecurity | Panic Buying | 1.000 | 0.628 | Retained |
| Perceived Food Insecurity | FIES Food Insecurity | 1.000 | 0.921 | Retained |
| Panic Buying | Budget Pressure | 1.000 | 0.863 | Retained |
| Panic Buying | Food Shopping Behaviors | 1.000 | 0.967 | Retained |
| Food Consumption Pattern | Food Shopping Behaviors | 0.996 | 0.747 | Retained |
| Food Consumption Pattern | Food Purchasing Motives | 1.000 | 0.789 | Retained |
| Food Consumption Pattern | FIES Food Insecurity | 1.000 | 0.698 | Retained |
| Food Shopping Behaviors | Food Purchasing Motives | 1.000 | 0.669 | Retained |
| FIES Food Insecurity | BAI Anxiety | 0.995 | 0.904 | Retained |
| Age Group | Education | 0.860 | 0.554 | Retained |
| Age Group | Chronic Disease | 0.938 | 0.620 | Retained |
| Age Group | Marital Status | 1.000 | 0.590 | Retained |
| Age Group | Employment | 1.000 | 0.545 | Retained |
| Employment | Gender | 0.984 | 0.657 | Retained |
| Employment | Income Adequacy | 0.953 | 0.680 | Retained |
| Gender | BMI | 0.996 | 0.850 | Retained |
| Gender | Alcohol | 0.984 | 0.612 | Retained |
| Marital Status | BMI | 0.981 | 0.885 | Retained |
| Alcohol | Smoking | 0.999 | 0.676 | Retained |
| Breakfast | Meal Skipping | 1.000 | 0.559 | Retained |
| Meal Skipping | Meals per Day | 0.904 | 0.623 | Retained |
| *Gender** | *Physical Activity** | *0.839* | *0.716* | *New at 0.70* |
| *Employment** | *Education** | *0.837* | *0.601* | *New at 0.70* |
| *Income Adequacy** | *EFINUB Food Cons. Impact** | *0.749* | *0.671* | *New at 0.70* |
| *Budget Pressure** | *FIES Food Insecurity** | *0.766* | *0.698* | *New at 0.70* |

*Strength = proportion of 1,000 bootstrap replicates in which the arc appeared; Direction = proportion of those replicates with the indicated direction. Retained = present in the primary model (S ≥ 0.85); New at 0.70 = admitted only at the relaxed threshold. * = arcs admitted at the relaxed threshold. Total: 25 retained + 4 new = 29 arcs.*

All 25 arcs from the primary model were retained in the relaxed network, confirming that the conservative threshold did not exclude arcs that would later prove unstable. The four newly admitted arcs predominantly involved cross-domain connections between demographic, lifestyle, and dietary habit variables. These additional connections suggested that at a lower threshold, the three previously isolated clusters began to exhibit inter-cluster connectivity, though the core scale-relationship pathway remained structurally invariant. Figure S1 displays the complete network at the 0.70 threshold, with solid lines representing retained arcs and dashed red lines indicating newly admitted arcs. The core pathway from EFINUB Food Consumption Impact through FIES Food Insecurity to BAI Anxiety remained unchanged across threshold specifications.


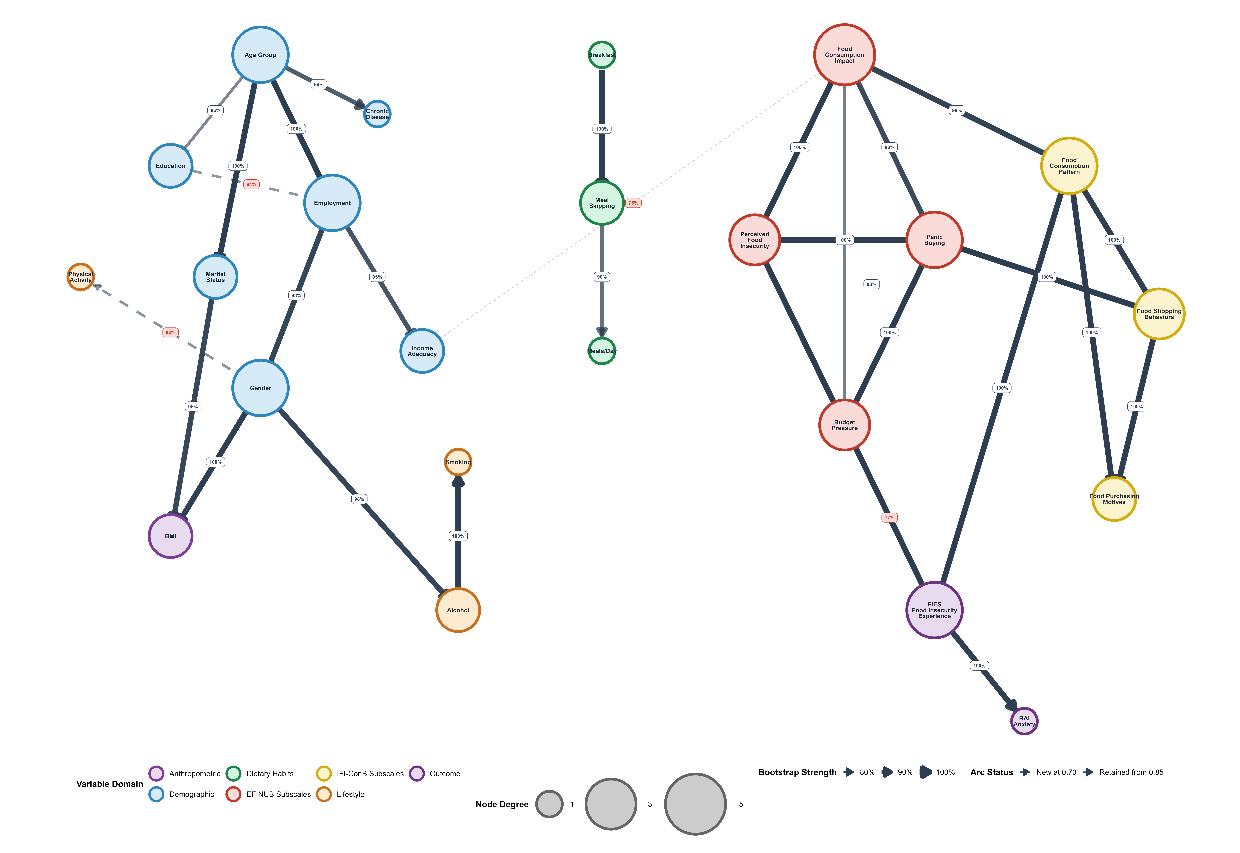


**Figure S1.** Bootstrap-averaged Bayesian Network at a relaxed strength threshold of S ≥ 0.70 (29 arcs). Solid lines indicate arcs retained from the primary model (S ≥ 0.85); dashed red lines indicate additional arcs admitted at the lower threshold. Node layout, coloring, and sizing conventions follow Figure 1 in the main text. The core pathway from EFINUB Food Consumption Impact through FIES Food Insecurity to BAI Anxiety remained structurally invariant across thresholds.

Figure S2 characterizes the distribution of bootstrap arc strength values. Panel A reveals a bimodal distribution with a gap around the 0.85 region, supporting this value as a natural threshold. Panel B illustrates the trade-off between network complexity and structural stability across seven candidate thresholds.


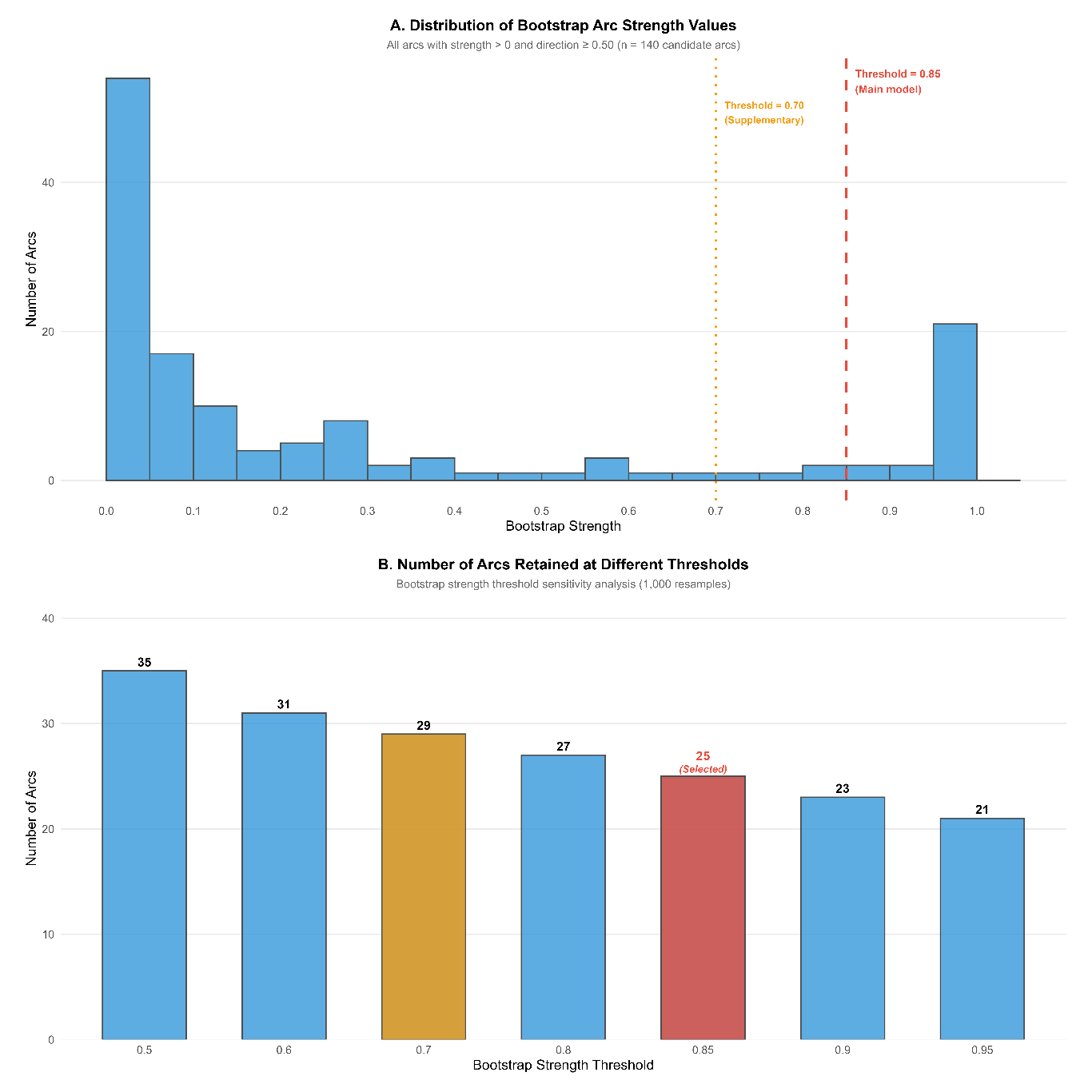


**Figure S2.** Bootstrap arc strength threshold sensitivity analysis. *(A) Histogram of bootstrap arc strength values for all candidate arcs with strength > 0 and direction ≥ 0.50. Vertical dashed lines indicate the primary threshold (0.85, red) and supplementary threshold (0.70, orange). (B) Number of arcs retained at seven candidate thresholds (0.50–0.95). The selected threshold of 0.85 (red bar) balanced network parsimony with structural completeness.*

# S6. Gender-Balanced Sensitivity Analysis

The original sample exhibited a gender imbalance (72.9% female, 27.1% male). A gender-balanced subsample (n = 564; 282 males, 282 females) was created by random undersampling of females, and the full bootstrap stability procedure (1,000 resamples with HC algorithm and BIC-CG scoring, model averaging at the 0.85 threshold) was repeated on this subsample, yielding 22 directed arcs. Table S7 presents the arc-by-arc comparison between the two networks.

**Table S7.** Comparison of directed arcs between the full-sample network (n = 1,042) and the gender-balanced network (n = 564) at the 0.85 threshold.

| **From** | **To** | **Full Sample (n=1,042)** | **Balanced (n=564)** | **Status** |
| --- | --- | --- | --- | --- |
| EFINUB Food Cons. Impact | Perceived Food Insec. | ✓ | ✓ | Shared |
| EFINUB Food Cons. Impact | Panic Buying | ✓ | ✓ | Shared |
| EFINUB Food Cons. Impact | Food Cons. Pattern | ✓ | ✓ | Shared |
| Perceived Food Insec. | Panic Buying | ✓ | ✓ | Shared |
| Perceived Food Insec. | FIES Food Insecurity | ✓ | ✓ | Shared |
| Panic Buying | Budget Pressure | ✓ | ✓ | Shared |
| Panic Buying | Food Shop. Behav. | ✓ | ✓ | Shared |
| Food Cons. Pattern | Food Shop. Behav. | ✓ | ✓ | Shared |
| Food Cons. Pattern | Food Purch. Motives | ✓ | ✓ | Shared |
| Food Cons. Pattern | FIES Food Insecurity | ✓ | ✓ | Shared |
| Food Shop. Behav. | Food Purch. Motives | ✓ | ✓ | Shared |
| FIES Food Insecurity | BAI Anxiety | ✓ | ✓ | Shared |
| Age Group | Education | ✓ | ✓ | Shared |
| Age Group | Marital Status | ✓ | ✓ | Shared |
| Age Group | Employment | ✓ | ✓ | Shared |
| Gender | BMI | ✓ | ✓ | Shared |
| Gender | Alcohol | ✓ | ✓ | Shared |
| Marital Status | BMI | ✓ | ✓ | Shared |
| Alcohol | Smoking | ✓ | ✓ | Shared |
| Breakfast | Meal Skipping | ✓ | ✓ | Shared |
| Meal Skipping | Meals per Day | ✓ | ✓ | Shared |
| Marital Status | Physical Activity | — | ✓ | Balanced only |
| EFINUB Food Cons. Impact | Budget Pressure | ✓ | — | Full only |
| Employment | Gender | ✓ | — | Full only |
| Employment | Income Adequacy | ✓ | — | Full only |
| Age Group | Chronic Disease | ✓ | — | Full only |
| Meal Skipping | Meals per Day | ✓ | ✓ | Shared |

*Shared = arc present in both networks; Full only = present only in the full-sample network; Balanced only = present only in the gender-balanced network. ✓ = arc present; — = arc absent. Jaccard similarity index: J = |Shared| / |Shared ∪ Full only ∪ Balanced only| = 21/26 = 0.808.*

The Jaccard similarity index of J = 0.808 indicated high structural agreement between the full-sample and balanced-sample networks. Of the 26 unique arcs across both networks, 21 were shared, 4 were present only in the full-sample model, and 1 was unique to the balanced model. Three key findings emerged from the comparison. First, 12 of the 13 arcs within the scale-relationship cluster (EFINUB and IFI-ConB subscales, FIES, BAI) were shared between the two networks, the core dependency pathway (EFINUB Food Consumption Impact → subscales → FIES → BAI) was structurally invariant. Additionally, 9 of the 13 demographic and dietary habit arcs were also shared, indicating that the network structure was highly stable across different gender compositions. Second, the four arcs present only in the full-sample network were EFINUB Food Consumption Impact → Budget Pressure (S = 0.878), Employment → Gender (S = 0.984), Employment → Income Adequacy (S = 0.953), and Age Group → Chronic Disease (S = 0.938). The non-recovery of these arcs was attributable to reduced statistical power (n = 564 vs. 1,042). Notably, the Budget Pressure node remained connected to the network through its shared parent arc from Panic Buying, indicating that only the direct influence from the EFINUB hub was attenuated, not the node's overall integration into the scale-relationship cluster. Third, the balanced model recovered one additional arc not present in the full-sample network: Marital Status → Physical Activity. This connection, which fell just below the bootstrap threshold in the primary analysis, became detectable in the gender-balanced subsample. The emergence of this single additional arc, compared to the four that were lost, further supported the interpretation that the primary network captured the essential dependency structure and that the gender imbalance did not introduce spurious connections. The high Jaccard index (J = 0.808) represented a substantial improvement over what would be expected from comparing networks of different sizes under random conditions, and confirmed that the substantive conclusions of the primary analysis—particularly the mediating role of food insecurity between inflation-related behavioral changes and psychological distress—were robust to the gender composition of the sample.

# S7. Computational Reproducibility

All analyses were conducted in R version 4.5.2 (R Core Team, 2025) on a Windows platform. A fixed random seed (set.seed(2026)) was used for all stochastic procedures, including bootstrap resampling, likelihood-weighted sampling, and gender-balanced random undersampling, to ensure full reproducibility of the reported results. Table S8 presents the complete list of R packages and their versions used in the analysis pipeline.

**Table S8.** R packages and versions used in the analysis

| **Package** | **Version** | **Purpose** |
| --- | --- | --- |
| bnlearn | 5.1 | BN structure learning, bootstrap stability, parameter estimation, Markov blanket identification, conditional probability queries |
| mclust | 6.1.2 | Gaussian Mixture Model analysis for data-driven categorization |
| psych | 2.5.6 | Internal consistency reliability (Cronbach’s alpha, KR-20) |
| igraph | 2.2.1 | Network object construction and graph metrics (degree, adjacency) |
| ggraph | 2.2.2 | Network visualization (ggplot2-based graph rendering) |
| ggplot2 | 3.5.2 | Statistical graphics, conditional probability panels, threshold sensitivity plots |
| patchwork | 1.3.2 | Multi-panel figure composition and layout |
| openxlsx | 4.2.8.1 | Data import from Excel files |
| RM.weights | 2.0 | Rasch (one-parameter logistic) modeling of the FIES: item parameters, fit statistics, reliability, respondent severity |
| parallel | 4.5.2 | Parallelized bootstrap resampling across 14 CPU cores |

*All packages were sourced from CRAN. R version 4.5.2 (2025-10-31 ucrt). The parallel package is part of the base R distribution.*

Key computational parameters were as follows: bootstrap resampling used R = 1,000 iterations with the Hill-Climbing algorithm and BIC-CG scoring, parallelized across 14 CPU cores; likelihood-weighted conditional probability queries used N = 50,000 samples per query with a fixed seed of 2026; the gender-balanced sensitivity analysis used the same bootstrap procedure (1,000 iterations, HC algorithm, BIC-CG scoring, threshold 0.85) on the undersampled subsample; and GMM model selection evaluated 1–6 components under both equal-variance and variable-variance structures using BIC optimization. The complete R analysis scripts and the anonymized dataset are available from the corresponding author upon reasonable request.

# S8. EFINUB Food Consumption Categorization Sensitivity Analysis

The primary Bayesian Network categorized the EFINUB Food Consumption subscale into three levels (Low, Moderate, High) using a Gaussian Mixture Model, while the remaining six continuous subscales were retained in their original metric (Section 2.4; Tables S3–S4). To evaluate whether this selective categorization influenced the learned network structure, a sensitivity analysis was conducted in which the EFINUB Food Consumption subscale was instead retained as a continuous variable. The full structure-learning and bootstrap stability procedure (1,000 resamples, Hill-Climbing algorithm, BIC-CG scoring, model averaging at the 0.85 threshold) was repeated on the otherwise identical 23-node dataset (n = 1,042), and the resulting network was compared arc-by-arc with the primary (GMM-based) network. Table S9 presents this comparison.

**Table S9.** Structural comparison of the primary network (EFINUB Food Consumption categorized via GMM) and a sensitivity network in which EFINUB Food Consumption was retained as a continuous variable (bootstrap-averaged, 1,000 resamples, threshold S ≥ 0.85)

| **Metric** | **Value** |
| --- | --- |
| Arcs, primary (GMM) network | 25 |
| Arcs, continuous-variable network | 26 |
| Shared arcs | 22 |
| Arcs unique to GMM network | 3 |
| Arcs unique to continuous network | 4 |
| Jaccard similarity index | 0.759 |
| Core pathway (EFINUB Food Consumption → … → FIES → BAI) | Preserved |
| Markov blanket of BAI, GMM network | FIES (single node) |
| Markov blanket of BAI, continuous network | FIES (single node) |
| Markov blanket of BAI, continuous network across thresholds (0.70 / 0.85 / 0.90) | FIES at all thresholds |

*Both networks were learned using the Hill-Climbing algorithm with BIC-CG scoring and bootstrap model averaging (1,000 resamples, S ≥ 0.85). The Jaccard index was computed on the union of directed arcs across the two networks, with the categorized node mapped to its continuous counterpart for matching. Arcs unique to the primary network: EFINUB Food Consumption → Panic Buying, Panic Buying → Budget Pressure, and Age Group → Education. Arcs unique to the continuous-variable network: EFINUB Food Consumption → FIES Food Insecurity, Budget Pressure → Panic Buying, Employment → Education, and Gender → Physical Activity.*

The two networks showed high structural concordance (Jaccard similarity = 0.759), with 22 of the 26 unique arcs shared between specifications—a level of agreement comparable to that observed in the gender-balanced sensitivity analysis (Section S6; J = 0.808). Critically, the entire core pathway linking EFINUB Food Consumption through perceived food insecurity and food consumption pattern to FIES Food Insecurity and ultimately BAI Anxiety was fully preserved in the continuous-variable network. Moreover, the continuous specification recovered an additional direct EFINUB Food Consumption → FIES Food Insecurity arc, indicating that the upstream behavioral-impact-to-food-insecurity relationship was, if anything, more pronounced when the subscale was modeled continuously. Most importantly, the Markov blanket of BAI Anxiety comprised FIES Food Insecurity alone in both specifications and remained invariant across all three bootstrap thresholds (0.70, 0.85, 0.90) in the continuous-variable network. The few discordant arcs were confined to peripheral connections unrelated to the central pathway, including the direction of the Panic Buying–Budget Pressure arc, the EFINUB Food Consumption → Panic Buying link (which became indirect via Perceived Food Insecurity in the continuous model), and selected demographic and lifestyle edges. Taken together, these results confirm that the GMM-based categorization of the EFINUB Food Consumption subscale enhanced interpretability—by providing discrete behavioral-impact strata for the conditional probability queries and policy scenarios—without materially altering the learned conditional dependency structure or the principal finding that food insecurity constitutes the sole conditional dependency of anxiety (Table S9).

**S9. Conditional Gaussian Assumption Diagnostics**

The Conditional Gaussian (CG) Bayesian Network framework (Section 2.5.2) assumes that each continuous node follows an approximately normal distribution conditional on its discrete and continuous parent configurations, rather than marginally. To assess the tenability of this assumption, the distributional properties of the eight continuous nodes were examined both marginally and conditionally on their parents in the learned network. For the conditional assessment, each continuous node was regressed on its network parents, and the skewness and excess kurtosis of the resulting residuals were computed. Table S10 presents both sets of diagnostics.

**Table S10.** Marginal and conditional distributional diagnostics for the eight continuous nodes.

| **Continuous node** | **Marginal skewness** | **Marginal excess kurtosis** | **Conditional (residual) skewness** | **Conditional (residual) excess kurtosis** |
| --- | --- | --- | --- | --- |
| EFINUB Perceived Food Insecurity | −0.00 | −0.97 | 0.42 | 2.28 |
| EFINUB Panic Buying | −0.14 | −0.91 | 0.69 | 1.30 |
| EFINUB Budget Pressure | 0.26 | −0.99 | −0.12 | 1.14 |
| IFI-ConB Food Consumption Pattern | 0.59 | −0.68 | 0.48 | 0.98 |
| IFI-ConB Food Shopping Behaviors | 0.13 | −1.13 | 0.53 | 0.47 |
| IFI-ConB Food Purchasing Motives | 0.30 | −1.08 | 0.69 | 1.73 |
| FIES Food Insecurity Experience | 0.18 | −1.35 | 0.22 | −0.08 |
| BAI Anxiety | 0.77 | −0.09 | 0.78 | 0.41 |

*Marginal statistics were computed on the observed continuous variables (n = 1,042). Conditional statistics were computed on the residuals obtained by regressing each continuous node on its parents in the learned network. Skewness and excess kurtosis are reported using the standard moment-based definitions. Thresholds of |skewness| < 2 and |excess kurtosis| < 7 are commonly regarded as acceptable for Gaussian-based and structural-equation models*

Marginal skewness was modest for all continuous variables (|skewness| ≤ 0.77), and the marginal distributions were characterized primarily by mild platykurtosis (negative excess kurtosis) rather than heavy tails, which represents the least problematic form of departure from normality for Gaussian-based estimation. More importantly, the conditional residuals—which reflect the distributional assumption actually imposed by the CG parameterization—were well-behaved across all nodes, with skewness between 0.12 and 0.78 and excess kurtosis between −0.08 and 2.28, all comfortably within conventionally accepted thresholds (|skewness| < 2, |excess kurtosis| < 7). The two continuous nodes on the principal pathway showed particularly favorable profiles: the residuals of FIES Food Insecurity (skewness = 0.22, excess kurtosis = −0.08) and BAI Anxiety (skewness = 0.78, excess kurtosis = 0.41) were close to Gaussian. Although Shapiro–Wilk tests were statistically significant for all nodes, this is an expected outcome at the present sample size (n = 1,042), at which such tests detect even trivial departures from normality; the magnitude of skewness and kurtosis is therefore the more informative indicator. Conditional Gaussian networks are additionally known to be robust to moderate non-normality of the kind observed here (Scutari & Denis, 2021). Taken together, these diagnostics indicate that the conditional Gaussian assumption is reasonably satisfied for the continuous nodes in the present model (Table S10).

**S10. FIES Rasch Model Validation**

The Food Insecurity Experience Scale was analyzed using the Rasch (one-parameter logistic) measurement model recommended by the FAO for FIES data, in which all eight items share a common discrimination (fixed at 1) and item severity parameters are estimated on a logit scale with the mean fixed at zero (FAO, 2020). The model was fitted to the eight dichotomous items (no missing responses) by conditional maximum likelihood using the RM.weights package (version 2.0; Cafiero, Viviani, & Nord, 2018), the analytical tool developed for FIES data analysis by the FAO Voices of the Hungry project. Equal weights were applied, as no post-stratification survey weights were used. Item fit was evaluated using infit and outfit mean-square statistics, with values approximately within 0.7–1.3 conventionally regarded as indicating adequate fit (Nord, 2014; FAO, 2020).

**Table S11.** Rasch model item severity parameters and fit statistics for the eight FIES items

| **Item (FAO label)** | **Severity (logit)** | **SE** | **Infit** | **Outfit** |
| --- | --- | --- | --- | --- |
| **WORRIED** — worried about food | -1.224 | 0.103 | 1.155 | 1.426 |
| **HEALTHY** — unable to eat healthy/nutritious food | -1.117 | 0.102 | 1.097 | 1.264 |
| **FEWFOODS** — ate only a few kinds of foods | -0.668 | 0.098 | 0.916 | 0.920 |
| **ATELESS** — ate less than thought should | -0.189 | 0.095 | 0.943 | 0.931 |
| **RANOUT** — household ran out of food | 0.022 | 0.095 | 1.018 | 1.008 |
| **SKIPPED** — skipped a meal | 0.319 | 0.096 | 0.826 | 0.787 |
| **HUNGRY** — hungry but did not eat | 1.247 | 0.106 | 0.919 | 0.900 |
| **WHLDAY** — went without eating for a whole day | 1.609 | 0.114 | 1.113 | 1.058 |

*Severity parameters are on a logit scale with the mean constrained to zero and a common discrimination of 1 (Rasch assumption). SE = standard error. Infit and outfit are mean-square fit statistics; values approximately within 0.7–1.3 indicate adequate fit. Items are ordered from least to most severe.*

The item severities followed the theoretically expected ordering, ranging from the least severe item (WORRIED, −1.224 logits) to the most severe (WHLDAY, +1.609 logits), consistent with the established global severity ordering of the FIES items (Cafiero et al., 2018). Infit mean-square values for all eight items fell within the conventionally accepted range (0.826–1.155), indicating that the items conformed to the equal-discrimination assumption of the Rasch model and functioned coherently as a unidimensional scale. Outfit values were within range for six items; the two least severe items, WORRIED (outfit = 1.426) and HEALTHY (outfit = 1.264), showed mildly elevated outfit, a pattern commonly observed for these items in FIES applications across countries and attributable to a small number of unexpected responses among otherwise food-secure respondents (Cafiero et al., 2018). As outfit is more sensitive to such isolated aberrant responses than infit, and as the corresponding infit values remained within range, these items were retained without affecting scale validity. The Rasch reliability (analogous to Cronbach's alpha) was 0.71, exceeding the FAO benchmark of 0.70; the classical Kuder–Richardson Formula 20 reliability for the same items was 0.886, both indicating acceptable internal consistency in this sample.

**Table S12.** Respondent severity (theta) and standard error by FIES raw score

| **Raw score** | **n** | **Theta (logit)** | **SE** |
| --- | --- | --- | --- |
| 0 | 265 | -3.059 | 1.487 |
| 1 | 91 | -2.257 | 1.107 |
| 2 | 80 | -1.323 | 0.870 |
| 3 | 83 | -0.640 | 0.796 |
| 4 | 120 | -0.024 | 0.781 |
| 5 | 105 | 0.601 | 0.808 |
| 6 | 99 | 1.313 | 0.891 |
| 7 | 44 | 2.291 | 1.128 |
| 8 | 164 | 3.118 | 1.487 |

*Theta = estimated respondent location on the latent food-insecurity severity continuum (logit metric) for each raw score; SE = standard error of the ability estimate (largest at the extreme scores, as expected). n = number of respondents at each raw score.*

Because the sample-specific Rasch metric was not equated to the FAO global reference scale, the prevalence figures reported in the main text (food secure, 0–3; moderate, 4–6; severe, 7–8) are based on raw-score cutpoints and are presented as a simplified approximation rather than as internationally calibrated, FAO-comparable prevalence estimates of moderate-or-severe and severe food insecurity (see Section 2.2 and the Discussion). The Rasch validation reported here confirms that the FIES items satisfied the measurement assumptions of the model in this sample, supporting the use of the FIES raw score as the food-insecurity variable in the Bayesian Network.

The Rasch model diagnostics for the FIES are summarized graphically in Figure S3, which presents the person–item targeting, item fit, item characteristic curves, and test information function across four panels.


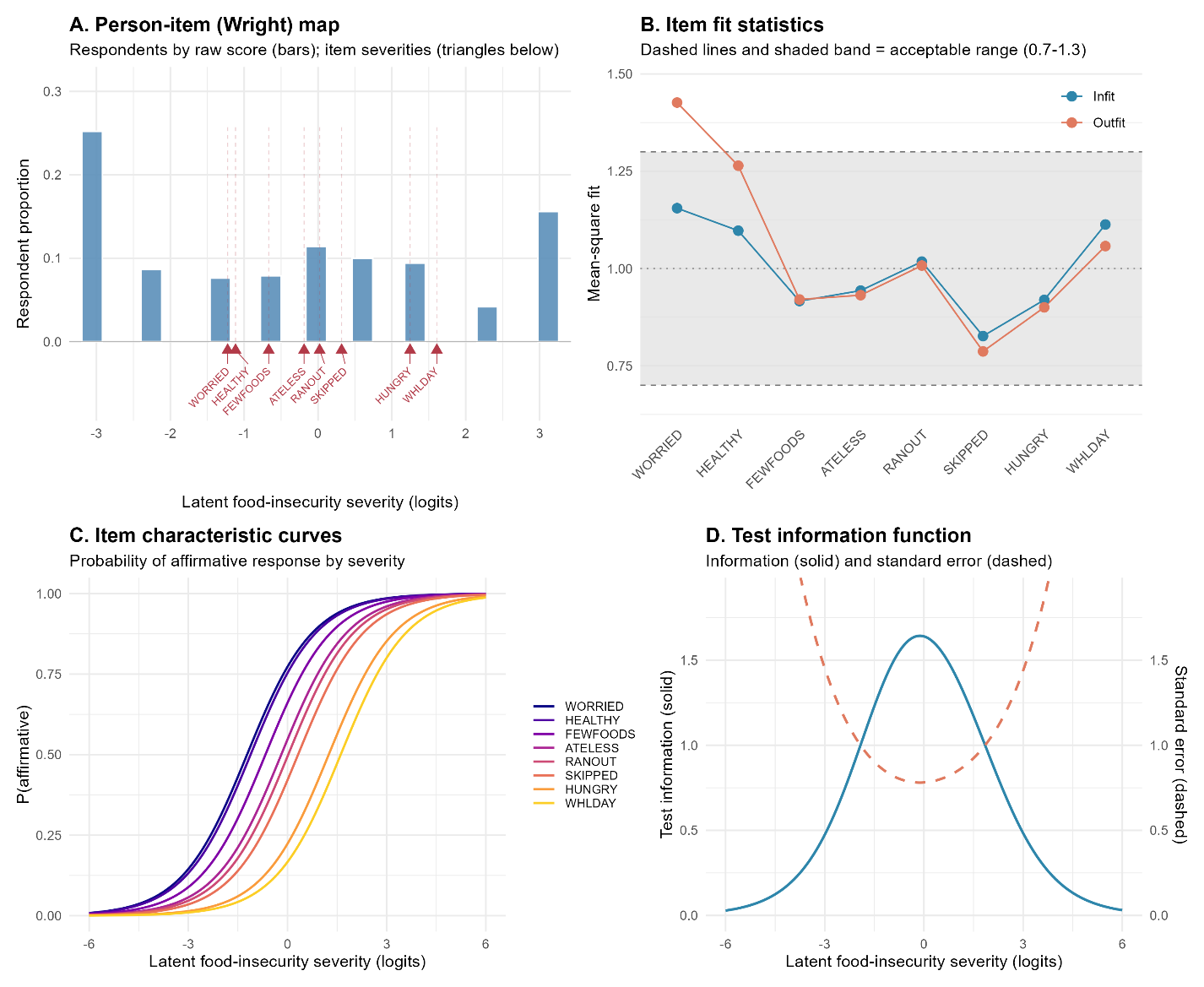


**Figure S3.** Rasch model diagnostics for the Food Insecurity Experience Scale. *(A) person–item Wright map, (B) item infit and outfit statistics, (C) item characteristic curves, and (D) test information function. Parameters were estimated by conditional maximum likelihood (RM.weights); panel B values correspond to Table S11.*

The person–item map (Panel A) shows that the eight item severities span a moderate range of the latent continuum (approximately −1.2 to +1.6 logits) and cluster near its center, while the respondent distribution extends more widely toward both extremes; this indicates that the scale is well targeted at respondents with mild-to-moderate food insecurity but provides less measurement precision at the lowest (fully food-secure) and highest (severely food-insecure) ends, where items are sparser. The item fit panel (Panel B) confirms that all items fell within or close to the conventional 0.7–1.3 range, with only the two least severe items (WORRIED, HEALTHY) showing mildly elevated outfit, consistent with the pattern commonly reported for these items in international FIES applications. The item characteristic curves (Panel C) display the expected parallel, non-crossing form predicted by the equal-discrimination assumption of the Rasch model, ordered from the least to the most severe item. Finally, the test information function (Panel D) peaks near the center of the latent scale (around 0 logits) and declines symmetrically toward the extremes, mirroring the targeting pattern in Panel A and indicating that the FIES measures food insecurity most precisely in the mid-range of severity. Taken together, these diagnostics support the unidimensionality, item ordering, and measurement adequacy of the FIES in the present sample.

# Supplementary References

Cafiero, C., Viviani, S., & Nord, M. (2018). Food security measurement in a global context: The Food Insecurity Experience Scale. *Measurement, 116*, 146–152.

Celeux, G., & Soromenho, G. (1996). An entropy criterion for assessing the number of clusters in a mixture model. *Journal of Classification, 13*(2), 195–212.

Food and Agriculture Organization of the United Nations. (2020). *Using the FIES app: A simple tool for the analysis of Food Insecurity Experience Scale data.* Rome: FAO.

Nord, M. (2014). *Introduction to Item Response Theory Applied to Food Security Measurement: Basic Concepts, Parameters and Statistics.* Rome: FAO.

R Core Team (2025). *R: A Language and Environment for Statistical Computing.* R Foundation for Statistical Computing, Vienna, Austria.

Scrucca, L., Fraley, C., Murphy, T. B., & Raftery, A. E. (2023). *Model-Based Clustering, Classification, and Density Estimation Using mclust in R.* Chapman & Hall/CRC.

Scutari, M., & Denis, J.-B. (2021). *Bayesian Networks: With Examples in R* (2nd ed.). CRC Press.

TURKSTAT (2024). *Address-Based Population Registration System Results.* Turkish Statistical Institute.
